# Designed Reactive Natural Deep Eutectic Solvents for Lipase-Catalyzed Esterification

**DOI:** 10.3390/molecules30040778

**Published:** 2025-02-07

**Authors:** Alina Ramona Buzatu, Anamaria Todea, Raluca Pop, Diana Maria Dreavă, Cristina Paul, Ioan Bîtcan, Marilena Motoc, Francisc Peter, Carmen Gabriela Boeriu

**Affiliations:** 1 Department of Applied Chemistry and Engineering of Organic and Natural Compounds, Faculty of Industrial Chemistry and Environmental Engineering, University Politehnica Timişoara, Carol Telbisz 6, 300001 Timisoara, Romaniaanamaria.todea@upt.ro (A.T.); diana.dreava@upt.ro (D.M.D.); cristina.paul@upt.ro (C.P.); ioan.bitcan@upt.ro (I.B.); francisc.peter@upt.ro (F.P.); 2 Department of Biochemistry and Pharmacology, Faculty of Medicine, “Victor Babes” University of Medicine and Pharmacy Timisoara, Eftimie Murgu Sq. no. 2, 300041 Timişoara, Romania; motoc.marilena@umft.ro; 3 Faculty of Pharmacy, “Victor Babeş” University of Medicine and Pharmacy Timişoara, Eftimie Murgu Square 2, 300041 Timişoara, Romania; pop.raluca@umft.ro; 4 Research Institute for Renewable Energies (ICER), University Politehnica Timisoara, Gavril Musicescu 138, 300501 Timişoara, Romania

**Keywords:** reactive NADES, lipase, immobilized lipase, catalytic activity, lipase stability, choline chloride, carbohydrates, molecular properties

## Abstract

Natural deep eutectic solvents (NADESs) are a sustainable, green option for extraction and reaction media in biorefineries and various chemical and biotechnological applications. Particularly, enzymatic reactions profit from NADES applications, as these solvents help to maintain high substrate solubility while improving both enzyme stability and efficiency. Recent studies confirmed that NADESs can perform multiple functions simultaneously, as reaction media for biocatalytic conversions, but also as substrates and catalysts for reactions, fulfilling the role of a reactive solvent. This study reports the beneficial effect of designed reactive natural deep eutectic solvents (R-NADESs) on the esterification activity and thermal stability of free and immobilized lipases in the synthesis of polyol- and carbohydrate-based biosurfactants. We manufactured and characterized 16 binary and ternary R-NADES systems with choline chloride (ChCl) as the hydrogen bond acceptor (HBA) and carbohydrate polyols; mono-, di-, and oligosaccharides; urea (U); N-methyl urea (MU); and water as the hydrogen bond donors (HBDs), in different combinations and molar ratios, most of which are reported for the first time in this paper. We determined their physicochemical, thermal, and molecular properties, including among others viscosity, polarizability, and the number of hydrogen bonds, and we showed that these properties are controlled by composition, molar ratio, molecular properties, temperature, and water content. Many lipases, both native and immobilized, showed high stability and remarkable catalytic performance in R-NADESs during esterification reactions.

## 1. Introduction

Natural deep eutectic solvents (NADESs) represent the third generation of deep eutectic solvents (DESs), according to the classification introduced by Abbott [1]. They are composed entirely of natural and biobased materials, with choline chloride (ChCl) and betaine as the hydrogen bond acceptor (HBA), and a large variety of polyols, carbohydrates, carboxylic acids, terpenes, amines, and amides as hydrogen bond donors (HBDs), interconnected with each other through hydrogen bonding in an extended network. A strong hydrogen bond network is the basis for NADESs’ unique physical, thermal, and mechanical properties [1,2]. A priori, scientists labelled NADESs as “green”, based on the intrinsic properties of their secondary metabolites’ constituents. Indeed, rigorous studies showed that NADESs are biodegradable and nontoxic for humans and living organisms, as well as biocompatible and environmentally benign [3,4]. Because of their green character and biological acceptance, NADESs are a valid alternative to the volatile organic solvents and ionic liquids used in biocatalytic conversions [5,6]. It is not surprising that the number of studies reporting on the properties, functionality, and the potential application of NADESs in various fields, including materials, the pharmaceutical industry [7,8], the cosmetic industry [9], and synthetic biocatalysis [10] increased significantly in the past years. It is relevant for the successful implementation of biocatalytic processes in NADESs as nonconventional media that many enzymes, for instance lipases, laccases, peroxidases, lyases, proteases, alcohol dehydrogenases, and oxidases, among others, are catalytically active and maintain stability, enantioselectivity and regioselectivity in a choline chloride-based NADES with ethylene glycol, glycerol, and urea, in an aqueous solution containing various amounts of water, ranging from 5 to 45% [11]. Therefore, recent research addressed the interaction between enzymes and NADESs, to better understand the relationship between the properties and components of the NADESs and the active structure and performance of enzymes [12,13].

Lipases are biocatalysts with the most applications in industries, both for (a) hydrolytic processes, in the food and laundry industries, and for (b) the synthesis of a large variety of esters via esterification, transesterification, interesterification, and (poly)condensation reactions in the fragrance and flavor, cosmetic, and pharmaceutical industries, as well as for the synthesis of biobased detergents, polymers, and biodiesel, among others [14,15,16]. Synthetic reactions usually proceed in organic solvents or solvent-free systems [17,18]. Already in 1984, A.M. Klibanov and his team showed that lipases function optimally in organic solvents as non-conventional media [19]. In the past two decades, lipases were proven to perform as well in ionic liquids and in both hydrophilic and hydrophobic DES and NADES systems [20,21,22,23]. The esterification of lactic acid with ethanol catalyzed by Novozyme 435 was explored in several DESs, the highest yield of 28.7% being obtained in choline chloride–glycerol (1:2) and 10% water content in a DES, at 50 °C [24]. Lipase-catalyzed esterification in DESs can be an effective route for the separation of racemic mixtures as well. Craveiro et al. obtained 44% conversion and an enantiomeric excess of 62% for the esterification of *rac*-menthol with lauric acid catalyzed by *Candida rugosa* lipase [25]. Fatty acid esters were produced from waste oil and ethanol in a DES (choline chloride–glycerol, 1:2) and the ultrasound-assisted DES systems, with immobilized lipase from *Candida antarctica* B (Novozyme 435) as a catalyst. The conversions reached 94% in ultrasonic conditions [26]. Lipoamino acids are difficult compounds to synthesize in classical reaction systems, but Nian et al. succeeded to obtain lauroyl glycine in an amidation reaction catalyzed by *Candida antarctica* B lipase in NADESs (with the best result obtained with a choline chloride–glycerin ratio of 1:2); this also showed that adding metal chlorides for three-constituent NADESs can increase their yield up to 86% [27]. Sugar esters represent another difficult synthetic task which was successfully approached by lipase-catalyzed processes in DESs. Noro et al. obtained high conversions of glucose laurate and glucose acetate by combining the appropriate DESs (choline chloride–urea; choline chloride–glycerol; or tetrabutylammonium bromide–imidazole) with lipases from *Aspergillus oryzae*, *Candida rugosa*, or a porcine pancreas [28].

Obviously, the chemical composition of a DES or NADES has a strong influence on the activity and catalytic efficiency of a lipase. Cao et al. [29] showed that the secondary structure of the *Candida antarctica* B lipase (CalB) strongly depends on the nature of the HBD component, as it is being preserved in alcohol-based NADESs, and denatured in carboxylic acid-based NADESs, consequently with similar effects on catalytic activity and thermal stability. The length of the carbon chain of the HBD component, the number of carboxyl groups in hydrophobic NADESs and the number of hydroxyl groups in hydrophilic NADESs had a positive impact on the catalytic efficiency and thermal stability of CALB, as demonstrated in the model reaction of benzyl alcohol acetylation [30]. ChCl-based NADESs with polyols as HBDs (i.e., glycerol, xylitol, D-arabitol, and D-sorbitol) maintain the active structure of the enzyme by substituting the hydration layer of the enzyme surface, thus protecting the enzyme at a higher temperature, and enhancing the reaction yield [30,31]. The higher catalytic efficiency of the CALB lipase in hydrophobic DESs compared to hydrophilic ones was confirmed by Hollenbach et al. in the synthesis of glucose monodecanoate [32], as the yields obtained in a hydrophobic DES consisting of (-)-menthol and decanoic acid were higher than previously reported for the hydrophilic DESs ChCl–urea and ChCl–glucose [33].

Reactive natural deep eutectic solvents (R-NADESs) detached themselves over the past years from the bigger and thoroughly studied group of NADESs due to their particular ability to play multiple functions when mixed with the proper catalyst, particularly enzymes. It is now well documented that, in addition to their role as a solvent for the reaction, R-NADESs can be (a) the source of a substrate, when one or both components of the R-NADES participate in the reaction, (b) a source of a catalyst, or (c) the initiator of a cascade reaction [34].

Despite the many scientific reports on the utilization and the effects of NADESs on the activity and thermal stability of a lipase when a NADES is used as the reaction medium, information on the effect of R-NADESs and their properties on lipase esterification activity when the solvent is also the source of the substrate of the reaction is scarce. Also, most studies reported in the literature were based on the hydrolytic activity of a lipase, using PNP hydrolysis as the model reaction, when the R-NADES was a co-solvent for aqueous systems [35] or for aqueous–organic solvent mixtures, such as DMSO and tert-butanol [36]. It is common knowledge that esterification activity and hydrolytic activities are quite different and could be divergent, since other factors are contributing, including the composition of the reaction medium and its physical and thermal properties, as well as the type, and the structure of the substrates and their interaction with the solvent and the enzyme [37]. This is particularly important for enzymes in R-NADES reaction systems, when the substrate of the reaction is a part of an extended hydrogen bond network surrounding the enzyme catalyst [33]. Moreover, the possibility of secondary reactions (the formation of choline chloride fatty esters) must be considered in the case of the esterification of fatty acids carried out in NADESs using choline chloride as the hydrogen bond acceptor and a sugar and water as the hydrogen bond donors, if free choline chloride, not involved in the H-bond network of the NADES, is present in the reaction system [38].

In this context, this study examined the effect of designed hydrophilic R-NADES mixtures on the esterification activity and thermal stability of free and immobilized lipases for the synthesis of polyol- and carbohydrate-based biosurfactants. The application of reactive NADES mixtures as the reaction medium and substrate source for lipase-catalyzed carbohydrate ester synthesis is of high importance to prevent a low substrate concentration caused by the limited solubility of carbohydrates in organic solvents and solvent-induced enzyme inactivation. We manufactured and characterized 16 binary and ternary R-NADES mixtures with ChCl as the HBA and carbohydrate polyols; mono-, di-, and oligosaccharides; urea (U); and N-methyl urea (MU) as the HBDs, in different combinations and molar ratios. Lipase enzymes were selected for application in an R-NADES based on their experimentally determined reference esterification activity for (a) the solventless synthesis of n-propanol laurate and (b) the synthesis of glucose-6-monolaurate, in DMSO/*t*-butanol 90/10 (vol%). The selected enzymes were immobilized by entrapment in a silica sol–gel matrix. All the native enzymes, the immobilized enzymes prepared, and the commercial CalB immobilized on acrylic resins (N435) were utilized as biocatalysts for the synthesis of the lauryl esters of the polyols and carbohydrates in the designed R-NADESs and the operational stability, esterification activity, and LA conversion were determined and evaluated in relation to the properties and composition of the used reactive NADES mixtures.

## 2. Results and Discussion

### 2.1. Preparation and Propeties of R-NADESs

**Figure 1:**
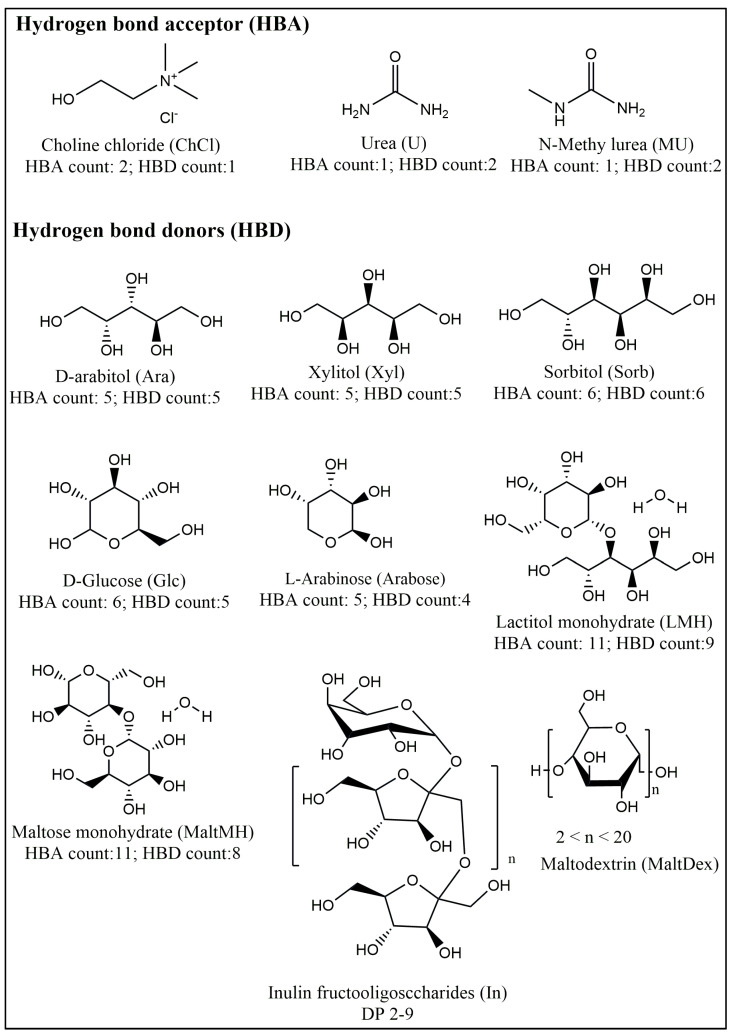
Structure of hydrogen bond donors (HBDs) and hydrogen bond acceptors (HBAs) used in this study for the preparation of reactive natural deep eutectic solvents (R-NADESs).

The R-NADESs were prepared by a direct mixing of the HBA component with the HBD components listed in Figure 1, at different molar ratios, as given in Table 1.

Choline chloride, with an HBA count of two, and urea and N-methyl urea, both with an HBA count of one, all can serve as an HBA, while U and MU, with an HBD count of two, can also function as HBD components. We prepared two sets of R-NADESs, namely (i) one set of binary mixtures containing only a carbohydrate or a polyol as the HBD with either ChCl (entries 1, 4, and 13–16 in Table 1) or U and MU as the HBDs (entries 6 and 7 in Table 1), and (ii) another set of ternary mixtures, with additional water (entries 2, 3, and 5 in Table 1) or with urea (entries 8–12 in Table 1) next to ChCl and the carbohydrate HBDs. The addition of water to the ChCl–carbohydrate ternary mixtures, (i.e., 5.5 wt.% to R-NADES 2, 3.8 wt.% to R-NADES 3, and 9 wt.% to R-NADES 5) was essential for the formation of deep eutectic solvents. During heating, the transparent liquid develops for all the mixtures (examples are in Figure S1 in the Supplementary Information file, SI). All prepared R-NADESs are clear, transparent liquids in the temperature interval between 40 and 80 °C, which is the optimum temperature range for lipase catalytic activity.

Computational chemistry and modeling gave us information on the interaction between the chemical components of the mixtures and the hydrogen bond network formation. A graphical representation of the structures of all 16 R-NADES mixtures is given in Figure S2, in the Supplementary Information file (SI). The H-bonds obtained, and their length are given in Table S1 in the SI, for all the R-NADES.

**Figure 2:**
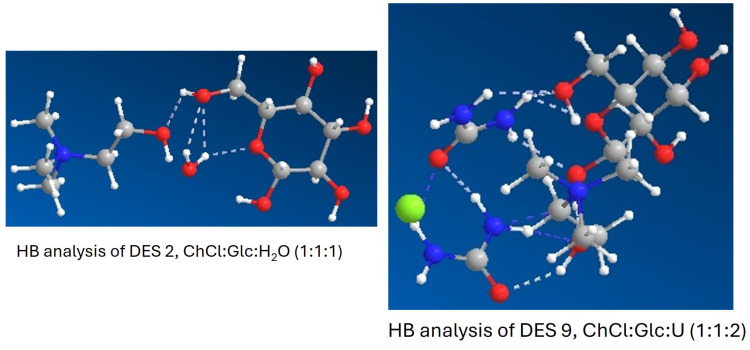
Hydrogen bond network and optimized structures of NADES 2, ChCl:Glc:H_2_O (1:1:1) and NADES 9, ChCl:Glc:U (1:1:2).

The optimized structures of NADES 2 (ChCl:Glc:H_2_O 1:1:1) and NADES 9 (ChCl:Glc:U 1:1:2) and the hydrogen bonds formed are illustrated in Figure 2. The visualization of the hydrogen bond network demonstrates without any doubt that all the constituents of R-NADES mixtures have multiple functions, each compound acting both as an HBD and as an HBA. In binary and ternary R-NADESs with carbohydrates and polyols, the interactions between the hydroxyl group of choline and the OH of carbohydrates and polyols, and the binding of water to the OH groups of carbohydrates are observed. In ternary R-NADES with ChCl, urea, and carbohydrates, there are multiple interactions between the NH_2_ groups of urea, as an HBD, and the -OH groups of the carbohydrate and choline chloride, but also between the carbonyl group of urea and the protons of the OH groups and of the amino groups, resulting in supramolecular structures. We can conclude that the number of hydrogen bonds increases with the number of components (NADES 10) and with the number of urea and methyl urea structures, which can easily establish intermolecular forces (NADESs 6, 7, and 9). The number of H-bonds created is determined by the composition of the mixtures and the stereochemistry of the molecules. This is clearly seen for the binary R-NADES mixtures composed of choline chloride and polyols. Xylitol-based R-NADESs, composed of meso-xylitol, i.e., (2R,3r,4S)-pentane-1,2,3,4,5-pentol, contain two H-bonds (Figure S2-14), while R-NADESs based on (a) D-arabitol, i.e.,(2R,4R)-pentane-1,2,3,4,5-pentanol (Figure S2-13), and (b) D-sorbitol, i.e., (2R,3R,4R,5S)-hexane-1,2,3,4,5,6-hexanol, (Figure S2-15), have only one H-bond.

#### 2.1.1. Thermal and Physical Properties of R-NADES Mixtures

The composition of the mixtures, the molar ratios, and the thermal properties, i.e., the onset temperature of decomposition (T_onset_), water loss, the melting temperature (T_m_), the glass transition temperature (T_g_), viscosity (η), are listed in Table 1. The thermograms of selected samples are in Figure S3, in the Supplementary file.

A thermogravimetric analysis (TGA) gave information on thermal stability, decomposition temperatures, and the free and bound water, providing detailed insight into the water interactions within the DES structures, and showing clear differences between the thermal properties. All binary and ternary ChCl-based R-NADES mixtures with polyol and carbohydrate HBDs (entries 1–5 and 13–16, Table 1) are highly thermostable, with T_onset_ ranging from 190 °C to 278 °C. They show minor weight loss up to 200 °C, which is mainly water. Only a small part of the crystallization water and the additional water in the ternary chloride–carbohydrate R-NADES mixtures (entries 1–5 in Table 1) are released at temperatures below 200 °C, which suggests that most of the water is tightly bound to other components in the extended hydrogen bond network. A major mass loss of about 80% was recorded between 200 and 300 °C. The R-NADES mixtures containing urea and MU (entries 6–12, Table 1) started decomposing at temperatures between 140 °C and 173 °C, which was caused by the thermal decomposition of urea and N-methyl urea, with a high mass loss of 20–45% at temperatures up to 200 °C. The low amounts of water in these mixtures are released at temperatures below 120 °C. The mixtures containing di- and oligosaccharides have increased thermal stability, irrespective of the other components and molar ratio.

A DSC analysis allowed us to follow the phase transition as a function of the temperature. The binary and ternary mixtures with urea and N-methyl urea (i.e., entries 6–12, Table 1) and the equimolar mixtures of ChCl:Glc:H_2_O, (1:1:1) (entry 5) are clear liquids when cooled and have a low glass transition temperature, ranging from –0.3 °C to −51.5 °C. They showed clean thermograms without thermal transitions in the positive temperature range. The DSC curves of ChCl:Xyl (1:1), ChCl:Ara (1:1), and ChCl:Sorb (1:1) (entries 13–16, Table 1) each present a distinct broad endothermic peak at 29.4 °C, 50.2 °C, and 44.2 °C, respectively, with the onset at around 2 °C for ChCl:Xyl (1:1) and 20 °C for the arabitol- and sorbitol-based mixtures. In the thermograms of the mixtures ChCl:Glc (2:1), ChCl:Glc:H_2_O (2:1:1), and ChCl:LMH (2:1) with an molar excess of ChCl, (entries 1, 3, and 16 in Table 1), the endothermic peaks observed at 76.9 °C, 73.7 °C, and 77.8 °C, respectively, could eventually be attributed to the excess ChCl not involved in the extended hydrogen bond network of the mixture. Aroso et al. [39] were the first to observe similar effects in the thermograms of mixtures of ChCl:Xyl at ratios of 4:1 and 3:1 and determined that the sharp endothermic peak at 78 °C for choline chloride, when it results from a crystallographic arrangement phase transition, is shifted to a lower temperature, around 75 °C, irrespective of the molar ratio. The DSC curve of the lactitol-based NADES (entry 16, Table 1) also shows a peak at 145 °C, which is due to the melting of excess lactitol, which is most probably dissolved in the NADES fluid but not bound in the hydrogen bond network. Ternary R-NADES mixtures also containing urea (i.e., entries 8–12, Table 1) and the equimolar mixtures of ChCl:Glc:H_2_O, (1:1:1) (entry 5 in Table 1) are clear liquids when cooled and have a low glass transition temperature, ranging from –0.3 °C to −51.5 °C. They show clean thermograms without thermal transitions in the positive temperature range, except for MU:Glc (2:1) (entry 7, Table 1), which shows transitions at 89.8 °C, which might be due to thermal degradation, as the discoloration also suggests. Examples of DSC thermograms are given in Figure S3.

**Figure 3:**
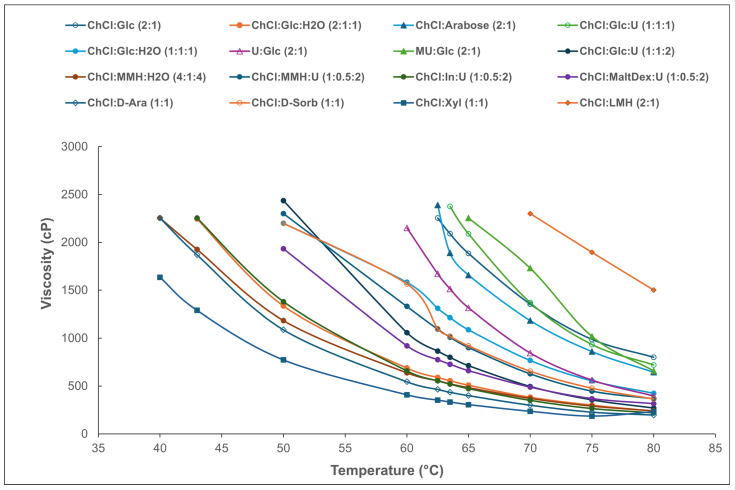
Viscosity (cP) of R-NADES mixtures as a function of temperature.

Rheological measurements allowed for the determination of the viscosity of all the R-NADESs (Figure 3). The results show the structure of the constituents of the R-NADES mixture, the molar ratio in which they are included, the water content, and the temperature, all of which have a strong effect on the fluid properties, and on viscosity. The extension of the hydrogen bond network, induced by the addition of water, decreases the mobility of the species in a solution, and the electrostatic and the van der Waals interactions contribute to the increase in viscosity. This is clearly seen from the 3-fold reduction in the viscosity of the almost dry ChCl:Glc (2:1) R-NADESs (entry 1 in Table 1) and by the addition of water ChCl:Glc:H_2_O (2:1:1) (entry 3 in Table 1). The nearly dry polyol-based R-NADESs (entries 13–15 in Figure 1) have the lowest values of viscosity compared to the carbohydrate-based and the urea-based mixtures, which have a higher number of and stronger intermolecular hydrogen bond interactions. However, the lactitol-based binary R-NADES (DES 16) is very viscous even at an elevated temperature, due to the high fraction of dispersed unbound choline chloride and lactitol. Based on experimental viscosity data, we observed the following increasing trend of viscosity as a function of the structure of the carbohydrate component:

ChCl/polyol < ChCl/monosaccharide < ChCl/disaccharide.

In all cases, the addition of water decreases viscosity. In the absence of water, deep eutectic mixtures cannot be obtained for disaccharides, like maltose. Nevertheless, R-NADESs with constitutive water embedded in the network during the preparation process are fundamentally different than the solutions obtained by adding an R-NADES as a co-solvent to water or a buffer, which is commonly used currently to reduce viscosity.

A Pearson correlation analysis showed only a weak relationship between the thermal properties that do not strongly predict viscosity (Figure S4, SI), suggesting that factors beyond these temperature-related properties influence viscosity at 70 °C. Overall, the lack of strong linear correlations between most of the properties suggests complex interactions, with each mixture showing unique behavior depending on its specific chemical composition (e.g., ChCl), highlighting the importance of considering chemical composition when interpreting the data.

Therefore, more insight into the relationship between the viscosity of R-NADESs and their composition and molecular properties was obtained by a principal component analysis (PCA). The parameters used for the PCA evaluation were the water content (% water), the viscosity, the molar ratio between the hydrogen bond acceptor (ChCl) and the corresponding hydrogen bond donors (HBA/HBDs), and the number of hydrogen bonds (HBs) in the NADES network determined by computational methods. R-NADES polydisperse mixtures containing maltodextrin (entry 12, Table 1) and inulin (entry 11, Table 1) were not included in this analysis, since their HB numbers could not be determined.

**Figure 4:**
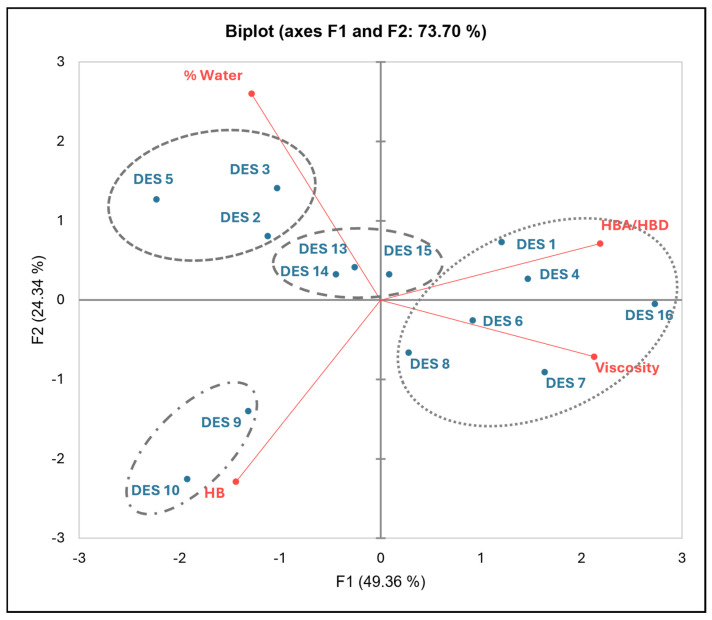
Relationship between the computed molecular properties of R-NADESs and their viscosity as determined by PCA.

The results clearly show the strong interdependency of the molecular properties of the R-NADES mixtures and their experimentally measured viscosity (Figure 4). There is a strong positive correlation between viscosity and the HBA/HBD ratio, and a negative correlation of viscosity with the water content and the H-bond numbers, respectively.

We identified four groups of R-NADESs that share comparable properties. Group 1 is composed of mixtures with a high viscosity, i.e., DES 1, DES 4, DES 6–8, and DES 16, all binary ChCl-based mixtures with a molar ratio, HBA/HBD, of two and with low water content. Group 2, containing ternary R-NADES carbohydrates and additional water, i.e., DES 2, DES 3, and DES 5, is characterized by mixtures with low viscosities and HBA/HBD ratios lower than one. Group 3 (DES 13, DES 14, and DES 15) and Group 4 (DES 9 and DES 10), although containing different compositions, are both characterized by mixtures with a low viscosity, despite the extremely low water content of these mixtures. The low viscosity of the R-NADES mixtures of Group 3, which are binary mixtures with ChCl as the HBA and with xylitol, arabitol, and sorbitol as the HBDs, can be associated with the equimolar ratio of HBA/HBD and the small number of hydrogen bonds. The ternary ChCl:U–carbohydrate mixtures of DES 9 and DES 10 in Group 4, are compact mixtures with high HB numbers and low HBA/HBD molar ratios.

To our best knowledge, this is the first time that the macroscopic fluid properties of R-NADESs, and of deep eutectic solvents in general, are correlated with the molecular properties of the mixtures. We think that this study could open the way for a more rational design of novel multifunctional R-NADES mixtures and will avoid the trial-and-error laborious experimental approach.

#### 2.1.2. Computational Characterization of R-NADES

**Figure 5:**
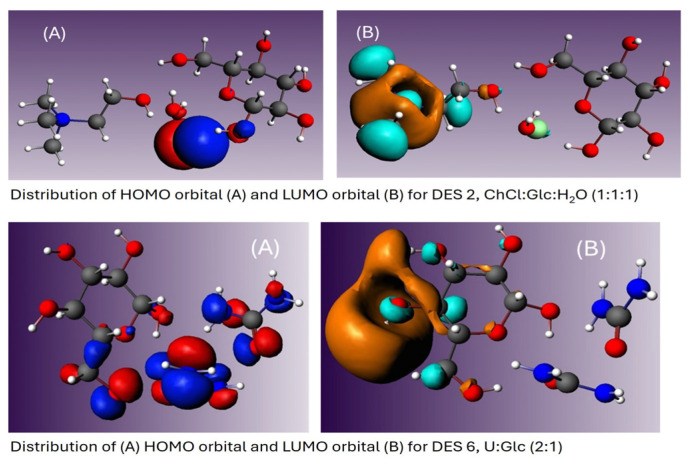
Distribution of HOMO and LUMO orbitals for NADES 2, ChCL:Glc:H_2_O (1:1:1) in the first line, and NADES 6, U:Glc (2:1), in the second line. Labels (**A**) refer to HOMO orbitals, and Labels (**B**) refer to LUMO orbitals.

Computational studies addressed the optimization of the HBA and HBD constituents of R-NADESs, and the determination of important parameters, like the total energies of both NADESs and their components, the energies of the frontier molecular orbitals HOMO and LUMO, polarizabilities, dipole moments, as well as steric parameters like the Connolly accessible Area (CAA) and Connolly Solvent-Excluded Volume (CSEV). The important parameters that reflect and explain the reactivity of an R-NADES mixture are listed in Table 2. Snapshots of the graphical distribution of the HOMO and LUMO orbitals for all the NADES mixtures are shown in Figure S5. The HOMO orbitals are localized at the chlorine atom of choline chloride, except for DES 6 and DES 7, which do not contain choline, where they are localized on the urea and methyl urea structure units. The LUMO orbitals are localized on the choline skeleton and, for DES 7 and DES 8, on the sugar moieties. Figure 5 below shows the graphical distribution of the HOMO and LUMO orbitals for R-NADES 6 (U:Glc 2:1) and R-NADES 2 (ChCl:Glc:H_2_O 1:1:1). The energies of the HOMO and LUMO molecular orbitals play a key role in stability and reactivity. Lower HOMO-LUMO gaps indicate the tendency for such a compound to be less stable and therefore more reactive. According to the calculated HOMO-LUMO gaps, the most stable (and less reactive) R-NADESs are proven to be R-NADES 6 (U:Glc 2:1) and R-NADES 7 (MU:Glc 2:1), the ones that do not contain choline chloride. The presence of a supplementary urea in the composition of a tertiary R-NADES mixture does not have a major influence on the global descriptors of the mixture, as suggested by the results obtained for R-NADES 8 and R-NADES 9, respectively. A major difference was observed for R-NADES 1 (ChCl:Glc 2:1), R-NADES 4 (ChCl:Arabose 2:1), and R-NADES 5 (ChCl:MMH:H_2_O, 4:1:4). According to the results, they are the least stable mixtures, characterized by similar values of the frontier molecular orbitals, thus leading to the lowest chemical potential and the highest electrophilic character which suggest increased reactivity. The largest values of polarizability and dipole moments have been obtained for the R-NADESs that have a higher ChCl molar content. Instead, lower values were obtained, as expected, for the binary urea-based NADESs 6 and 7, but also for the NADESs 13, 14, and 15, which contain equimolar amounts of ChCl and five- or six-carbon chain polyols.

### 2.2. Evidence for the Performant Use of the R-NADES as a Solvent and Substrate Source for Lipase-Catalyzed Esterification Reactions

The new concept for the enzymatic synthesis of the fatty acid esters of carbohydrates and carbohydrate polyols using a reactive NADES as the multifunctional reaction medium requires we obtain answers to several questions: (i) a selection of the enzymes with the highest esterification activity, (ii) an evaluation of the effects of a NADES’s composition and properties on esterification activity and the operational stability of free and immobilized enzymes, and (iii) an estimation of the synthetic capacity of the selected enzymes for the synthesis of the fatty acid esters of carbohydrates and carbohydrate polyols in a reactive NADES.

**Figure 6:**
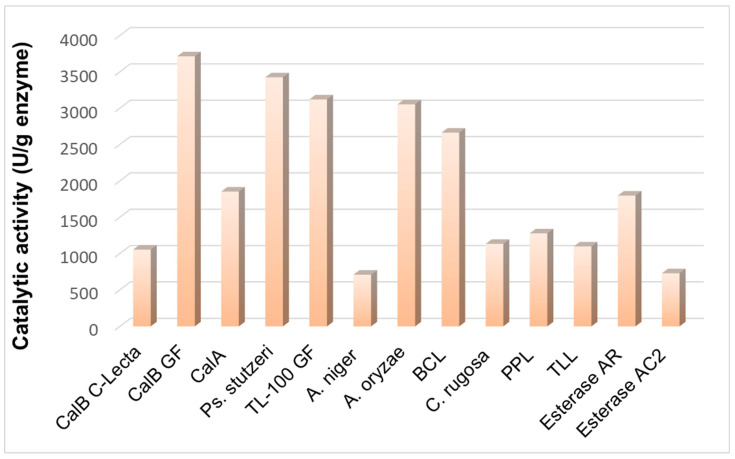
Esterification activity of native lipases during solvent-free esterification of lauric acid (LA) with n-propanol. Conditions: 55 °C; 1:1 molar ratio of LA/n-propanol; and 20 mg lipase/mL.

**Figure 7:**
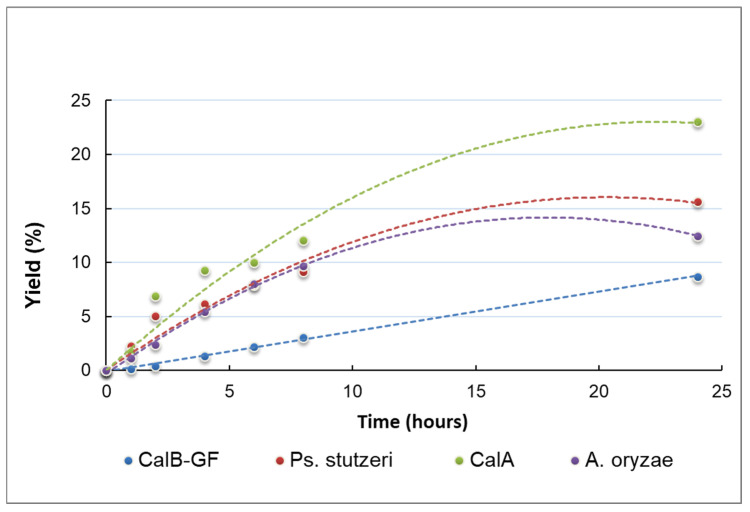
Time course for the synthesis of glucose laurate in 10% DMSO/90% *t*BuOH, at 70 °C, a 1:1 molar ratio of Glc/lauric acid, and 50 units of enzyme.

Thirteen commercial native lipases from different microbial sources were screened to select those with the highest esterification activity for (i) the synthesis of propyl laurate in a solventless system, and (ii) the synthesis of glucose laurate in a conventional solvent system. The progress of the reaction was followed by the quantification of the consumption of lauric acid during that time. All the tested enzymes were efficient in the solvent-free synthesis of propyl laurate (Figure 6). However, only a few enzymes, i.e., the lipases from *Candida antarctica* B (CalB), *Candida antarctica* A (CalA), *Pseudomonas stutzeri* (*Ps. stutzeri*), and *Aspergillus oryzae* (A. oryzae), also showed significant catalytic activity for glucose esterification (Figure 7). The formation of the glucose esters was confirmed by the TLC, HPLC, and MALDI-TOF-MS analyses.

**Figure 8:**
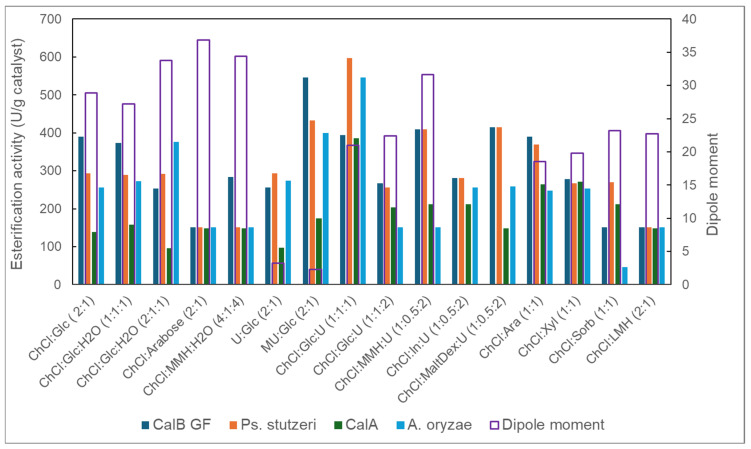
Catalytic activity (U/g enzyme) of selected native lipases for the esterification of n-propanol with lauric acid, at 70 °C, in reactive NADES mixtures.

The above-mentioned four enzymes showed significant esterification activity in all 16 binary and ternary reactive NADESs investigated, although they were lower than their reference esterification activity in a solventless reaction system (Figure 8). The R-NADES mixtures had a higher viscosity than the mixture of propanol with lauric acid used in the reference reaction, which can decrease the diffusion and the collision of the molecules, thus reducing the reaction rate. We observed that esterification activity was also influenced by the composition of the mixtures and the type of enzyme. CalB and *Ps. stutzeri* showed relatively high activity in all the ternary NADES mixtures. The CalB, *Ps. Stutzeri*, and CalA enzymes showed the highest catalytic activity in the ternary equimolar ChCl:Glc:U (1:1:2) NADES.

The results presented in Figure 8 demonstrate without any doubt the strong intrinsic relationship between the catalytic activity of the native lipase and the polarity of the R-NADES mixtures, represented by the dipole moment of the mixtures. All of the four enzymes tested showed the highest esterification activity in the R-NADES mixtures with the lowest dipole moments, i.e., the binary mixtures, R-NADES 6 (U:Glc 2:1) and R-NADES 7 (MU:Glc 2:1), without choline chloride. Overall, the lipases from *Candida antarctica* (CalB GF) and from *Pseudomonas stutzeri* (Ps. stutzeri) had the highest catalytic activity in all the tested R-NADES mixtures.

Moreover, the selected enzymes showed a relatively high thermal stability in the NADESs at 70 °C. The highest stability was observed in the first 24 h for the native *A. oryzae* and CalA lipases. However, considerable inactivation occurred after 72 h at 70 °C. CalA was the most stable, with 50% residual activity, followed by *A. oryzae*, *Ps. stutzeri*, and *CalB GF* with 30%, 20%, and 15% residual activity, respectively.

**Figure 9:**
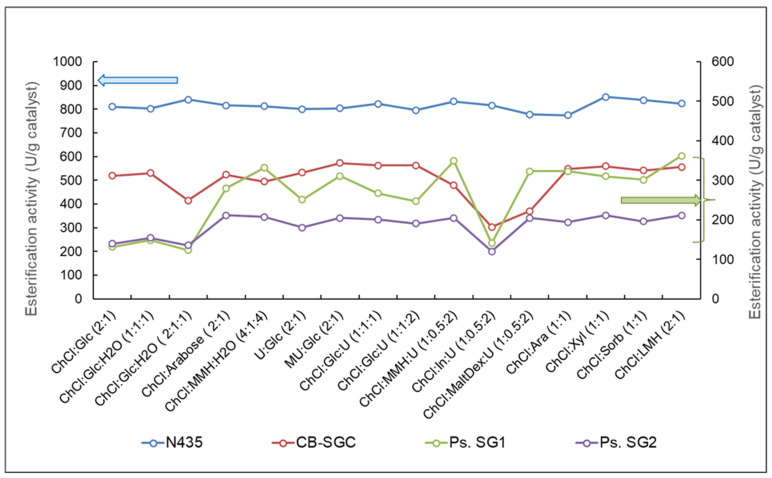
Catalytic activity (U/g enzyme) of the immobilized enzymes N435, CB SGC (CalB-GF immobilized using the silane precursors OcTMOS:TMOS 1:1 + adsorption on Celite), Ps. SG1 (*Ps. stutzeri* immobilized in sol–gel with OcTMOS:TMOS 1:1), and Ps. SG2 (*Ps. stutzeri* immobilized in sol–gel with 3-GoPrTMOS:TMOS 1:1), for the solventless esterification of n-propanol with lauric acid in R-NADES mixtures, at 70 °C, at 1:1 molar ratio, and with 50 U lipase/mL.

Since the thermal stability of enzymes can be considerably increased by immobilization on solid support, the native CalB-GF and *Ps. stutzeri* enzymes, which are known for responding well to immobilization, were immobilized by entrapment in silica sol–gel systems with or without combined adsorption on Celite. These at situ immobilized enzymes and the commercial CalB immobilized on acrylic resins, referred as N435, were evaluated to determine their catalytic activity and thermal stability in the NADESs. All immobilized enzymes showed remarkable esterification activity (Figure 9). The commercial CalB lipase immobilized on acrylic resins, N435, showed the highest esterification activity by far, with minor variations between the different R-NADESs used.

Among the enzymes immobilized by entrapment in silica sol–gel matrices, the lipase B from *Candida antarctica*-GF (labelled as CB-SGC in Figure 9) was the most active. The *Ps stutzeri* lipase immobilized with OcTMOS:TMOS (1:1) as silane precursors showed higher activity than the biocatalyst obtained with 3-GoPrTMOS:TMOS (1:1), probably due to the better permeability of the sol–gel matrix for the substrates when octyl pendant groups were present in its structure. The sol–gel immobilized enzymes showed a comparable pattern of activity, with minimal variation from R-NADES 4 to R-NADES 10 and R-NADES 12 to R-NADES 15, and lower levels of activity in R-NADES 3 and R-NADES 11.

**Figure 10:**
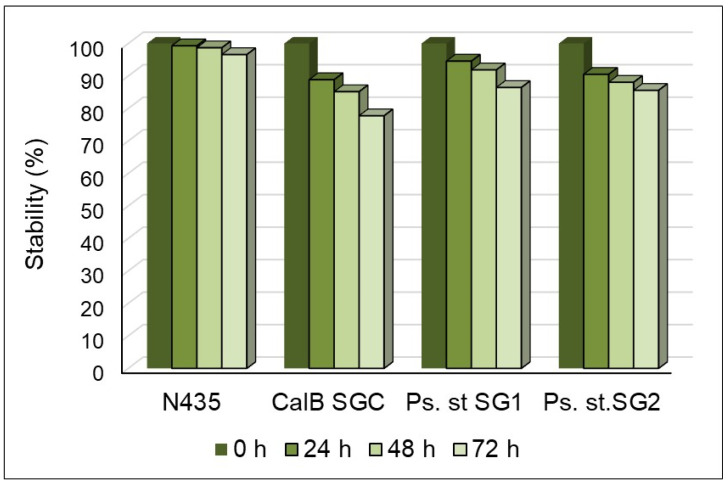
Thermostability of the immobilized enzymes in R-NADES at 70 °C.

All the immobilized enzymes were thermostable (Figure 10). The most stable was the commercial immobilized CalB, i.e., N435, with less than 10% activity loss after 72 h of mixing in a glucose–ChCl-based NADES at 70 °C. An insignificant loss of activity was observed after multiple cycles of mixing under similar conditions. The CalB-GF was immobilized on a silica sol–gel matrix, referred to as CalB-SGC, was less stable, losing approximately 22% of its activity under the same conditions. Both of the immobilized *Ps. stutzeri* lipases lost about 13.5% of activity.

**Figure 11:**
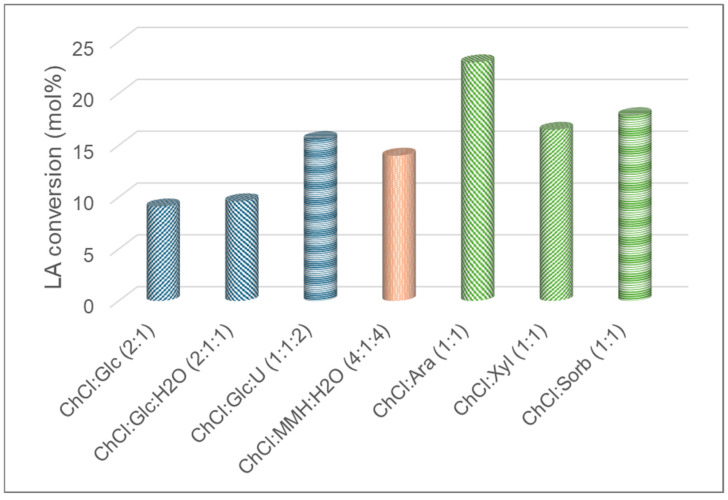
Substrate conversion during esterification of carbohydrates and carbohydrate polyols with immobilized CalB lipase (N435), in reactive NADESs, at 70 °C.

Ultimately, we explored the potential of the selected NADES mixtures with polyols and carbohydrates to fulfil a double function as the reaction medium and substrate in esterification reactions catalyzed by immobilized CalB (N435). The R-NADES mixtures showing the highest levels of enzyme’s thermostability and esterification activity were selected. The enzymatic synthesis of the lauryl esters of mono- and disaccharides and sugar polyols in the selected reactive NADESs was carried without any further addition of a carbohydrate or polyol as a substrate. Only lauric acid (LA) and an enzyme catalyst were added to each R-NADES, and the reaction mixtures were incubated for 72 h at 70 °C, with mixing. We tested three R-NADESs with glucose, R-NADES 1 ChCl:Glc (2:1), R-NADES 3, ChCl:Glc:H_2_O (2:1:1), and R-NADES 9, ChCl: Glc:U (1:1:2); one NADES with a disaccharide, R-NADES 5, ChCl:MMH:H_2_O (4:1:4); and three NADESs with polyols, R-NADES 13, ChCl:Ara (1:1), R-NADES 14, ChCl:Xyl (1:1), and R-NADES 15, ChCl–sorbitol (1:1). The progress of the reaction was monitored by HPLC, measuring the decrease in the concentration of lauric acid over time. The formation of mono-lauryl and di-lauryl esters of the carbohydrates and polyols in the corresponding ChCl/sugar or ChCl/polyol reactive NADESs was demonstrated by HPLC, MALDI-TOF-MS, and NMR. The type and properties of the R-NADES mixtures have a strong influence on the progress of a reaction, as shown for glucose esterification when the highest LA conversion (16%) was obtained for the low viscosity ChCl:Glc:U (1:1:2) R-NADES (Figure 11). The highest overall conversion (23%) was obtained for the arabitol lauryl ester, while the lowest conversion yields were obtained for the choline chloride-rich binary and ternary glucose-based R-NADES 1 ChCl:Glc (2:1) and R-NADES 3 ChCl:Glc:H_2_O (2:1:1). These results confirm that the tested R-NADES mixtures do play a double role as a reaction medium and substrate source and are genuine reactive NADES systems.

**Figure 12:**
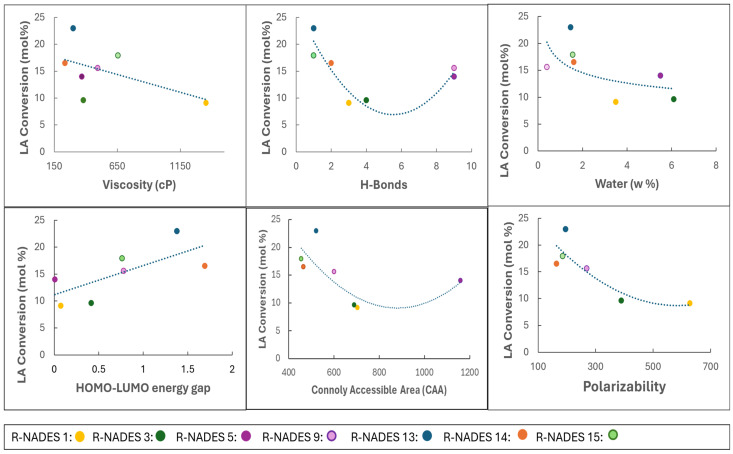
Correlation between physical and molecular properties of R-NADES mixtures and the efficiency of the enzymatic esterification of the carbohydrate and polyol components of the reactive NADESs, catalyzed by immobilized CalB, i.e., N435.

The Pearson correlation analyses show the intrinsic relationship between the physical and molecular properties of R-NADES mixtures and the efficiency of the lipase-catalyzed esterification of the carbohydrate and polyol components of the bifunctional R-NADESs studied, expressed as the molar conversion of the lauric acid substrate, referred to as the “LA Conversion” (Figure 12). LA conversions were the highest in the R-NADES mixtures with a low viscosity, i.e., lower than 650 cP, and decreased with an increase in viscosity. Similarly, the highest LA conversions were obtained in the R-NADES mixtures with a water content below 2 w% and decreased with an increase in the water content, reaching an equilibrium above 4 w%, most likely due to the reverse hydrolysis of the esters produced by the enzyme. This suggests that in such conditions an equilibrium between esterification and ester hydrolysis is preferred, despite the significant decrease in viscosity of the R-NADES mixtures due to dilution. The computed molecular properties of R-NADES mixtures define the efficiency of the reaction as well. There is a logarithmic relationship between the LA conversion and the number of H-bonds and the Connoly accessible area (CAA), which are strongly correlated with each other. Higher LA conversions were obtained in the reactions conducted in the R-NADESs with a lower H-Bond number (HB < 4) and a CAA < 800, which were less compact, and decreased with increases in both the CAA and the number of H-bonds. The observed increase in the LA conversion for the reactions in R-NADES 5 and R-NADES 9, each with higher CAA and H-Bond values, might be due to their low viscosity and the high water content of R-NADES 5, which might decrease the rigidity and compactness of the mixtures. As expected, the LA conversion decreased with an increase in the polarizability of the R-NADES mixtures and increased with the HL energy gap, due to the increase in reactivity.

## 3. Materials and Methods

### 3.1. Materials

Choline chloride (≥98%) and all polyols and carbohydrates, (i.e., D-arabitol (≥99%), D-sorbitol (≥98%), lactitol (≥99%), D-glucose (99.5%), D-arabinose (98%), D-maltose monohydrate (≥98%, 5–5.5% water), sucrose (99.5%), fructooligosaccharides (>90%, DP 2–9), maltodextrin (DE 15–20), urea (≥99%), methyl urea (≥99%), lipase B from *Candida antarctica*, immobilized on acrylic resin (N435), Porcine pancreas lipase (PPL), Amano lipase PS, from *Burkholderia cepacia*, *Candida rugosa* lipase AYS (Amano), TL lipolase AL from *Thermomyces lanuginosus*, octyltrimethoxysilane (OcTMOS), tetramethoxysilane (TMOS), and 3-glycidoxypropyltrimethoxysilane(3-GoPrTMOS), were purchased from Sigma-Aldrich (St. Louis, MO, USA). *Aspergillus niger* lipase and phenolphthalein were from Fluka (Seelze, Germany). Other enzymes used were lipase B from *Candida antarctica* purchased from C-Lecta, Leipzig, Germany (CalB C-Lecta); CalB-GF and *Thermomyces lanuginosus* lipase (TL-100 GF), which were a kind gift from GenoFocus (Daegeon, Republic of Korea), lipase A from *Candida antarctica* (CaLA) purchased from Chiralvision (Den Hoorn, Netherlands); *Pseudomonas stutzeri* lipase, a generous gift of Meito Sangyo, Nagoya, Japan (Ps. stutzeri); and *Aspergillus oryzae* lipase (Amano Enzyme, Nagoya, Japan). Dimethyl sulfoxide (DMSO), methanol, potassium hydroxide (KOH), hexane, and tetrahydrofuran (THF) were purchased from Merck (Darmstadt, Germany). Xylitol, 99%, lauric acid (LA), 98% were purchased from Thermo Scientific (Ward Hill, MA, USA). 1-Propanol and ethanol were from purchased Chimreactiv SRL (Bucharest, Romania). All chemicals were of analytical grade and were used as purchased, without further purification.

### 3.2. Methods

#### 3.2.1. Preparation and Characterization of R-NADESs

The preparation and characterization of binary and ternary R-NADES systems was conducted with the methods developed earlier by our group [31]. Eight binary and ten ternary reactive NADESs were prepared in a parallel reactor (STEM Omni Reaction Stations—Electrothermal, Thermo Scientific, Ward Hill, MA, USA) by mixing an HBA component, (i.e., ChCl, urea, or N-methyl urea) and each of the carbohydrate polyols, xylitol, D-arabitol, and D-sorbitol, as well as lactitol and carbohydrates, i.e., D-glucose, D-maltose monohydrate, sucrose, and inulin fructooligosaccharide, as HBDs, at different molar ratios, with or without stoichiometric addition of water, at 100 °C and 800 rpm, until homogenous fluid mixtures were obtained. After cooling at room temperature, the R-NADESs were analyzed to determine thermal and rheological properties. R-NADES mixtures were stored in sealed flasks in a dry environment, at room temperature.

Thermal data were collected using an TG-209-F1 Libra TG analyzer Netzsch (NETZSCH-Geraetebau GmbH, Selb, Germany), Netzsch Proteus-Thermal Analyzes version 6.1.0. (NETZSCH-Geraetebau GmbH, Selb, Germany). The conditions were as follows: nitrogen atmosphere, temperature range between 30 and 700 °C, and 10 K/min heating rate.

The R-NADES mixtures were characterized by DSC (DSC 204 F1 Phoenix differential scanning calorimeter, (NETZSCH-Geraetebau GmbH, Selb, Germany)) under a nitrogen atmosphere, in the temperature range of −80 °C to 150 °C, and a heating rate of 10 K/min.

Dynamic viscosities (η) were determined on a Brookfield rotational viscometer (DV2TRV, Ametek Brookfield (AMETEK, Inc., Berwyn, PA, USA), using the stainless-steel spindle type SC4-21, for temperatures ranging from 40 °C to 80 °C. Viscosities are given in cP, (cP = 1 mPa.s).

#### 3.2.2. Computational Details

The constituents of NADESs, the hydrogen bond acceptors and donors, were optimized at the BLYP/TZP [40] level of theory implemented in ADF2014 software [41]. The investigated NADESs were built using the previously obtained minima structures (the molar ratio is given in Table 1) and re-optimized through molecular mechanics. For the newly obtained NADES structures, single-point computations at the BLYP/TZP level of theory were performed. Parameters like the total energies of both the NADESs and their components, the energies of the frontier molecular orbitals HOMO and LUMO, polarizabilities [42], dipole moments, and the distribution of the frontier orbitals were calculated by means of the ADF2014 software. The steric parameters like the Connolly accessible Area, the Connolly Solvent-Excluded Volume, ovality, and the visualization of the hydrogen bonds established among the NADES components were determined with Chem 3DPro software, implemented in ChemBioDraw Ultra 14 [43].

The global reactivity descriptors, chemical potential (*µ*), chemical hardness (*η*), and electrophilicity index (*ω*), were calculated using Equations (1)–(3):

(1) $$\mu =\frac{E_{HOMO}+E_{LUMO}}{2}$$

(2) $$\eta =\frac{E_{LUMO}-E_{HOMO}}{2}$$

(3) $$\omega =\frac{\mu^{2}}{2\eta }$$

#### 3.2.3. Determination of the Esterification Activity of Free and Immobilized Enzymes

The esterification activity of native and immobilized lipases was determined for the synthesis of propyl laurate in (a) a solvent-free system, which served as reference activity, and (b) in the R-NADESs prepared, based on initial rate methodology [30]. Lipase activity was expressed in activity units, Us, where 1 unit of an enzyme is the amount of propyl laurate (µmol) produced per minute by 1 g of the enzyme (µmol · min^−1^ · g_enzyme_^−1^). In the solvent-free reaction system, equimolar amounts of n-propanol (0.4 g, 2 mmol) and lauric acid (LA; 150 μL, 2 mmol) were incubated with 10 mg of native enzyme, at 55 °C in 2 mL capped vials, stirred at 350 rpm in a thermomixer (Thermomixer Comfort, Eppendorf, Hamburg, Germany). The determination of lipase activity in R-NADESs followed the same approach, incubating LA (0.4 mmol) and 0.4 mmol of n-propanol with 0.8 g of NADES and an amount of enzyme corresponding to 50 reference activity units, at 70 °C and 1000 rpm. Esterification was monitored by the titration of the residual LA with an ethanolic KOH 0.1 M solution, with phenolphthalein as an endpoint indicator. All measurements were conducted in triplicate, and the mean value of the activity and the standard deviation are given.

#### 3.2.4. Lipase Immobilization

Immobilized lipases were obtained by entrapment in the sol–gel matrix, using standardized methods developed by our group [44], as follows:

Method SG1: To a 390 μL lipase solution (100 mg/mL) in a TRIS buffer with pH 8, 100 μL of PEG, 50 μL of NaF, and 100 μL of isopropanol were added. After homogenization, a mixture of the 3-GoPrTMOS and TMOS silane precursors was added at a 1:1 molar ratio and mixed until gelation. *Ps. stutzeri* lipase was immobilized using two mixtures of silanes: OcTMOS:TMOS 1:1 (Ps. SG1) and 3-GoPrTMOS:TMOS 1:1 (Ps. SG2).

Method SG2: This procedure was like method SG1, with the difference being that 250 mg of Celite was added after gelation. CalB-GF lipase was immobilized using a mixture of OcTMOS:TMOS 1:1, with the addition of Celite.

#### 3.2.5. Determination of the Thermal Stability of Native and Immobilized Lipases

Both native and immobilized lipases were incubated in each R-NADES for 0, 24, 48, and 72 h. Each time, the catalytic esterification activity of the enzyme was determined for the synthesis of n-propyl laurate, using the standard assay described at Section 3.2.2.

#### 3.2.6. Synthesis of Lauryl Esters of Polyols and Glucose with Immobilized Lipase in R-NADESs

Lauric acid, 1.6 mmol, and an amount of R-NADES equivalent to 1.6 mmol of a polyol or carbohydrate substrate were mixed in 5 mL of Eppendorf, and the reaction was initiated by the addition of 300 U/carbohydrate of immobilized CalB (N435). The reaction mixture was incubated for 67 h at 70 °C and 600 rpm in a thermomixer. The reaction was stopped by the addition of 5 mL of dimethyl sulfoxide, and after the separation of the catalyst, the mixture was analyzed by HPLC to determine the LA conversion and product formations.

#### 3.2.7. Characterization of the Esterification Products

The HPLC analysis used an Agilent System HPLC 1260 INFINITY II chromatograph (Agilent Technology, Waldbronn, Germany). The conditions were as follows: Kinetex 5 μm Polar C18 100 Å, 250 × 4.6 mm column; an eluent methanol/water ratio of 90:10; a flow rate of 0.2 mL/min; 50 °C; and a duration of 50 min. OpenLab CDS Workstation Software (version 2.8, Agilent Technology, Waldbronn, Germany) was used for visualization of chromatograms. The LA conversion (mol%) was calculated based on the calibration curve of LA.

High-resolution ^1^H and ^13^C NMR spectra were recorded on a Bruker Avance III spectrometer (Bruker Daltonics GmbH, Bremen, Germany) operating at 500 MHz, interfaced with a workstation running a Windows operating system and equipped with the TopSpin 3.5 software package. CDCl_3_ was used as a solvent. Chemical shifts (1H) are given in parts per million (ppm) and referenced to the solvent signals and to TMS.5.

## 4. Conclusions

In conclusion, in this study a large spectrum of reactive NADES mixtures with the physical and fluid properties suited for biocatalysis were successfully obtained and evaluated as reaction media for the synthesis of the lauric esters of monosaccharides, disaccharides and carbohydrate polyols. The native and immobilized lipase enzymes showed significant esterification activity and high thermal stability in the R-NADESs and were able to efficiently convert carbohydrates and polyols into their lauryl esters. The results are very promising and open the way to further exploring the potential of R-NADESs as a reaction medium for the enzymatic synthesis of the long alkyl chain esters of oligosaccharides.

By integrating molecular modeling, computational chemistry, and a thermophysical analysis with enzymology, we showed the strong relationship between the molecular and physical properties of R-NADESs and the esterification efficiency of free and immobilized lipases. Several studies discuss the influence of molecular properties on the physical properties of deep eutectic solvents [45,46], or on the solubility of carbohydrates in natural deep eutectic solvents [47], focusing on the application of DESs and NADESs as reaction mixtures. In this paper, we went one step forward, investigating the more complex reactive NADES systems and their interactions with enzymes.

The work reported here is an exploratory study, aiming to achieve a better understanding of the essential relationships between the structure and properties of R-NADESs and the catalytic activity, stability, and efficiency of enzyme catalysts. We achieved medium/low yields, due to the lower enzyme load used in these screenings, and the reactions were not optimized. Nevertheless, the reaction yields can be increased to industrially relevant levels through the optimization of the reaction conditions, with an emphasis on enzyme load, temperature, and reaction time; we demonstrated this through the lipase-catalyzed synthesis of carbohydrate polyol esters carried out in the choline chloride/polyol mixtures when we achieved product yields above 90 mol% [31].

The results demonstrate that R-NADESs are a promising category of natural reactive solvents that can be the basis of the development of greener synthetic biocatalysis for the synthesis of biobased chemicals, ingredients for food, and for pharmaceutical, biomedical, and technical applications. However, such a development requires a good understanding of (a) the properties and functionality of an R-NADES mixture, (b) the specific properties and requirements of enzymes to function in R-NADESs, and (c) the tools to achieve these targets.

## Figures and Tables

**Table 1:** Reactive NADES: composition and properties.

R-NADES		TGA		DSC		Viscosity at 70 °C (cP)	HBA/HBD ^#^	H-Bonds (HBs)
Entry	Composition	Water Loss (w%)	T_onset_ (°C)	T_m_ (°C)	T_g_ (°C)		(mol/mol)	
1	ChCl:Glc (2:1)	3.5	196	76.8	−52.3	1354	2	3
2	ChCl:Glc:H_2_O (1:1:1)	5.5	190	* n.o.	−44.5	767.5	0.5	4
3	ChCl:Glc:H_2_O (2:1:1)	6.08	195	8.7, 73.7	−22	382	1	3
4	ChCl:Arabose (2:1)	1.46	195	79.3	−20	1183	2	2
5	ChCl:MMH:H_2_O (4:1:4)	5.46	212	60.5	* n.o.	370	0.8	9
6	U:Glc (2:1)	0.86	142	89.4	−0.3	845	2	5
7	MU:Glc (2:1)	0.72	141	62.4	−0.4	1730	2	7
8	ChCl:Glc:U (1:1:1)	0.58	150	* n.o.	−27.5	1370	0.5	3
9	ChCl:Glc:U (1:1:2)	0.40	145	* n.o.	−28.5	495	0.33	9
10	ChCl:MMH:U (1:0.5:2)	1.28	153	* n.o.	−51.5	627.5	0.4	16
11	ChCl:In:U (1:0.5:2)	1.73	153	* n.o.	−51.5	350	0.4	^^^ n.d.
12	ChCl:MaltDex:U (1:0.5:2)	1.80	150	* n.o.	−51.5	487.5	0.4	^^^ n.d.
13	ChCl:D-Ara (1:1)	1.46	276.6	50.2	−62.1	300	1	1
14	ChCl:Xyl (1:1)	1.61	277.5	29.4	−58.6	235	1	2
15	ChCl:D-Sorb (1:1)	1.56	278	44.2	−55.7	655	1	1
16	ChCl:LMH (2:1)	1.14	245	61, 77.8, 145	* n.o.	2300	2	1

* n.o. = not observed; HBA/HBD ^#^ is the molar ratio between choline chloride and the sum of urea and carbohydrate or polyol as HBDs; ^ not determined.

**Table 2:** HOMO and LUMO energies, steric parameters, polarizability, and dipole moment.

R-NADES		E HOMO (H)	E LUMO (H)	HL Gap (eV)	Steric Parameters		Polarizability (a.u.)	Dipole Moment (D)
Nr.	Composition				CAA (Å2)	CSEV (Å3)		
1	ChCl:Glc (2:1)	−0.0825	−0.0798	0.073	703.831	489.463	627.690	28.903
2	ChCl:Glc:H_2_O (1:1:1)	−0.1109	−0.0858	0.682	578.348	316.263	192.431	27.248
3	ChCl:Glc:H_2_O (2:1:1)	−0.1064	−0.0911	0.416	689.734	488.310	389.509	33.830
4	ChCl:Arabose (2:1)	−0.0954	−0.0946	0.021	663.381	452.222	420.004	36.802
5	ChCl:MMH:H_2_O (4:1:4)	−0.0855	−0.0852	0.008	1160.43	1100.00	*	34.357
6	U:Glc (2:1)	−0.2141	−0.0320	4.953	484.641	246.967	168.796	3.204
7	MU:Glc (2:1)	−0.1938	−0.0275	4.523	532.677	288.432	181.260	2.254
8	ChCl:Glc:U (1:1:1)	−0.1003	−0.0774	0.622	640.500	358.293	256.863	21.018
9	ChCl:Glc:U (1:1:2)	−0.1121	−0.0834	0.781	600.765	419.292	269.003	22.417
10	ChCl:MMH:U (1:0.5:2)	−0.1314	−0.0857	1.243	957.111	869.306	*	31.588
13	ChCl:D-Ara (1:1)	−0.1311	−0.0804	1.379	521.383	288.194	195.500	18.497
14	ChCl:Xyl (1:1)	−0.1352	−0.0730	1.692	465.509	180.247	164.222	19.817
15	ChCl:D-Sorb (1:1)	−0.1168	−0.0888	0.762	455.295	214.224	185.314	23.195
16	ChCl:LMH (2:1)	−0.1128	−0.0817	0.846	657.739	458.275	288.993	22.689

* Polarizability computation for R-NADES 5 and R-NADES 10 failed, due to the large number of atoms.

## Data Availability

Data is available on request from the corresponding author.

## Supplementary Materials

The following supporting information can be downloaded at https://www.mdpi.com/article/10.3390/molecules30040778/s1: Figure S1: Images of selected R-NADES solvents at different temperatures; Figure S2: Hydrogen bonds analysis of the optimized structures; Table S1: Donors, acceptors, and hydrogen bond length within the investigated R-NADES; Table S2: Global reactivity descriptors of R-NADES; Figure S3: Thermograms, TG, and DSC of selected R-NADESs; Figure S4: Thermogravimetric properties of R-NADES mixtures and their relationships; and Figure S5: Graphic representation of the frontier molecular orbitals HOMO and LUMO distribution.
