# Supplementary material for: Designed Reactive Natural Deep Eutectic Solvents for Lipase-Catalyzed Esterification
Source: Molecules. 2025 Feb 7;30(4):778. doi: 10.3390/molecules30040778 (PMC11858590; doi:10.3390/molecules30040778)
Supplement: Supplementary file 1 [file molecules-30-00778-s001.zip › molecules-3428111-supplementary.pdf]

Supplementary information (SI)

Designed reactive natural deep eutectic solvents enhance esterification activity and thermal stability of lipases.

Alina Ramona Buzatu[1,2], Anamaria Todea[1], Raluca Pop[3], Diana Maria Dreavă[1], Cristina Paul[1], Ioan Bîtcan[1], Marilena Motoc[2], Francisc Peter[1,4], and Carmen Gabriela Boeriu[1,*]

[1] *Department of Applied Chemistry and Engineering of Organic and Natural Compounds, Faculty of Industrial Chemistry and Environmental Engineering, Polytechnic University of Timişoara, Carol Telbisz 6, 300001 Timisoara, Romania*

[2] *Department of Biochemistry and Pharmacology, Victor Babes University of Medicine and Pharmacy of Timisoara, Eftimie Murgu Sq. no. 2, 300041 Timişoara, Romania*

[3] *Faculty of Pharmacy, Victor Babes University of Medicine and Pharmacy of Timişoara, Eftimie Murgu Square 2, 300041 Timişoara, Romania*

[4] *Research Institute for Renewable Energies (ICER), University Politehnica Timisoara, Gavril Musicescu 138, 300501 Timişoara, Romania*

*E-Mail for CGB: carmengabriela.boeriu@upt.ro

Table of Contents

Contents

Figure S1. Images of selected R-NADES solvents at different temperatures. 3

Figure S2. Hydrogen bond analysis of the optimized R-NADES structures 4

Table S1. Donors, acceptors and hydrogen bond length within the investigated R-NADES 9

Figure S3. Thermograms, TG and DSC, of selected R-NADES. 10

Figure S4. Thermogravimetric properties of R-NADES mixtures and their relationships 11

Figure S5: Graphic representation of the frontier molecular orbital HOMO and LUMO orbitals for R0NADES...12

Table S2. Global reactivity descriptors of R-NADES...17

Figure S1. Images of selected R-NADES solvents at different temperatures.

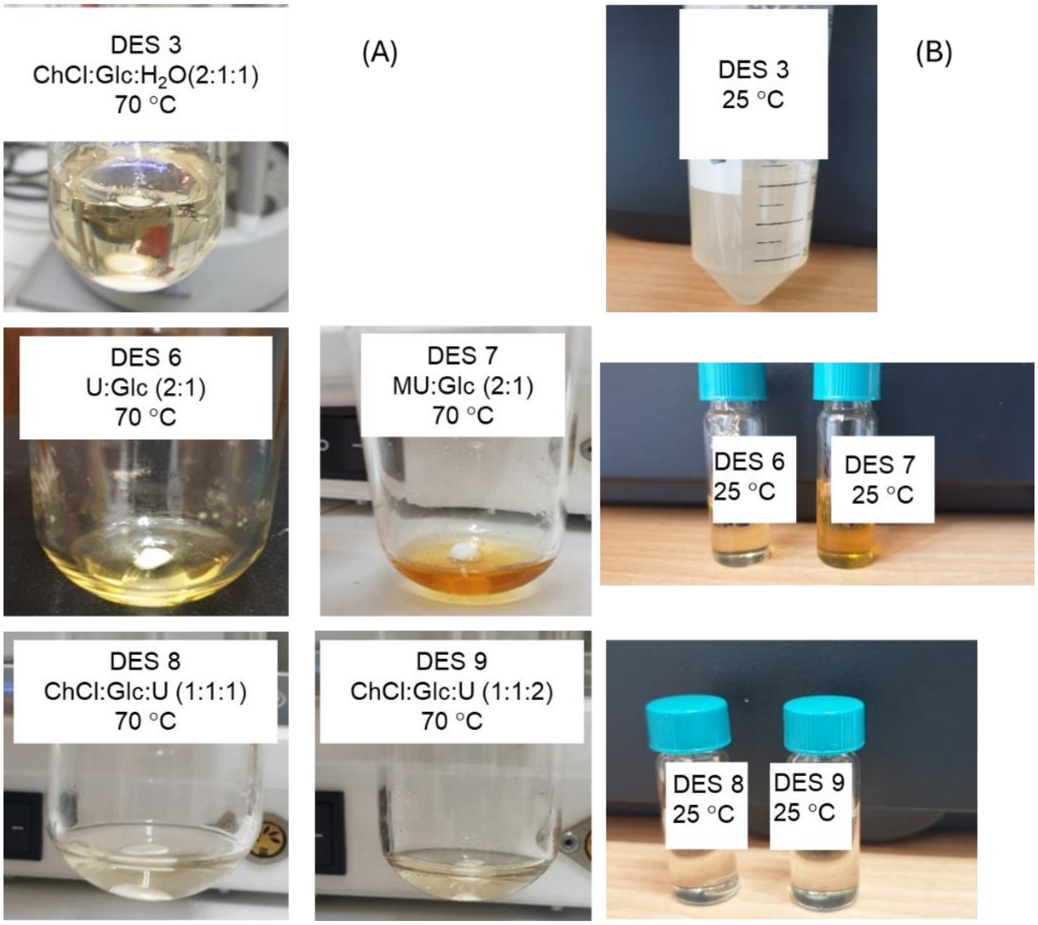

Figure S2. Hydrogen bond analysis of the optimized R-NADES structures

Figure S2-1. *HB analysis of DES 1, ChCl:Glc (2:1)*

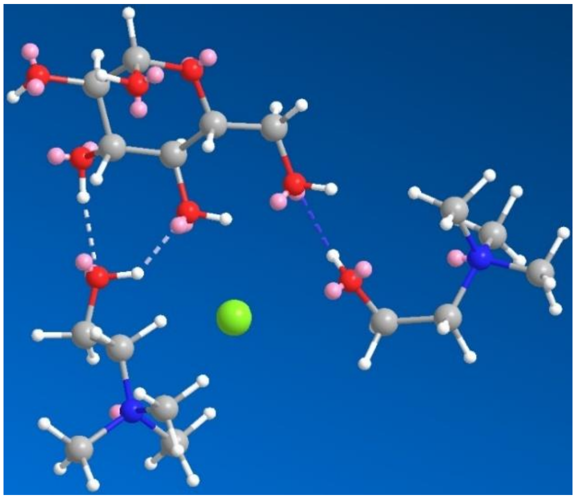

Figure S2-2. *HB analysis of DES 2, ChCl:Glc:H_2O (1:1:1)*

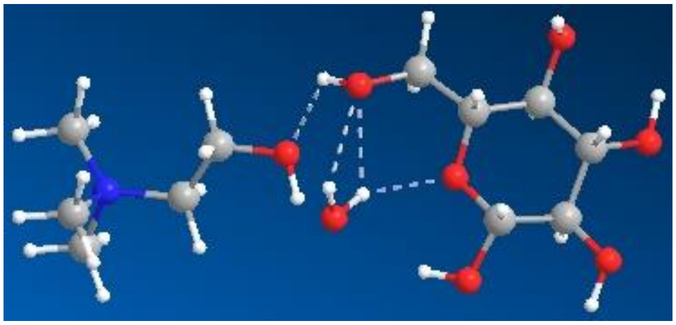

Figure S2-3. *HB analysis of DES 3, ChCl:Glc:H_2O (2:1:1)*

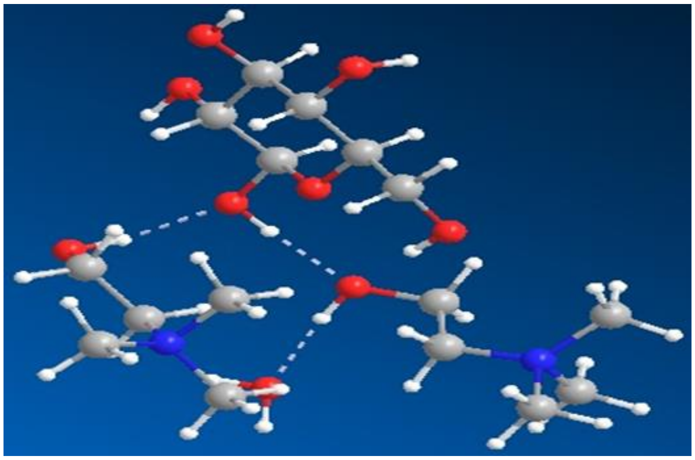

Figure S2-4. *HB analysis of DES 4, ChCl:Arabose (2:1)*

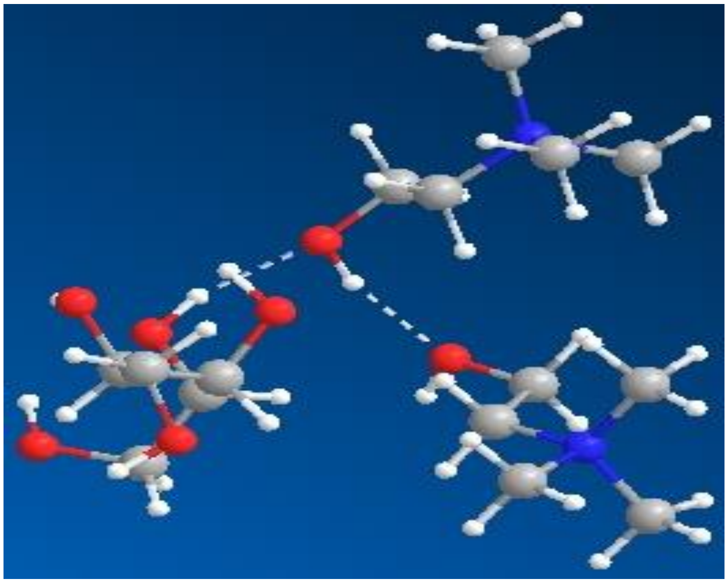

Figure S2-5. *HB analysis of DES 5, ChCl:MMH:H_2O (4:1:4)*

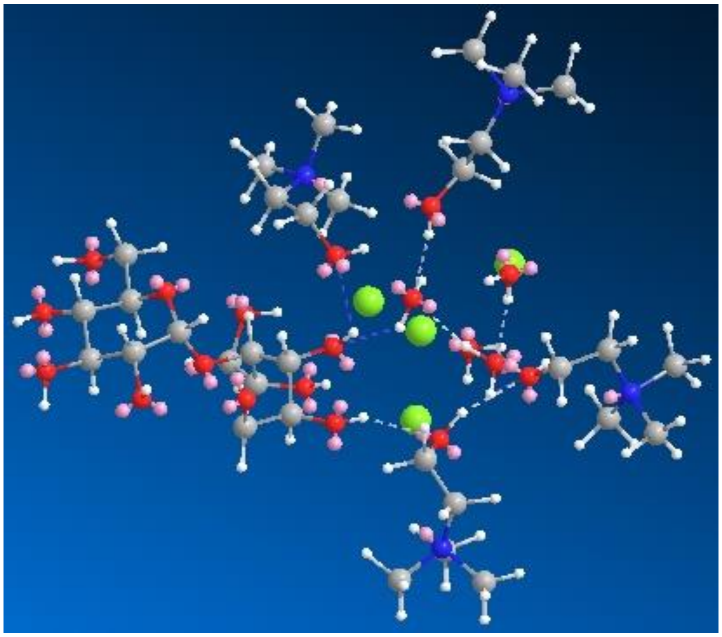

Figure S2-6. *HB analysis of DES 6, U:Glc (2:1)*

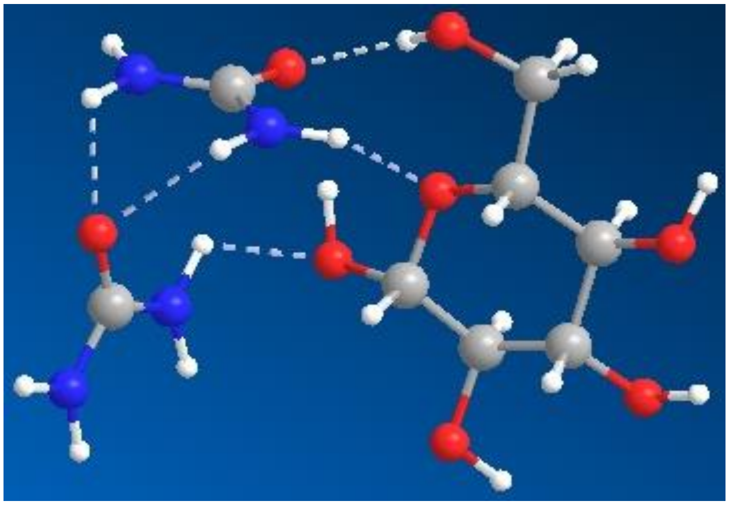

Figure S2-7. *HB analysis of DES 7, MU:Glc (2:1)*

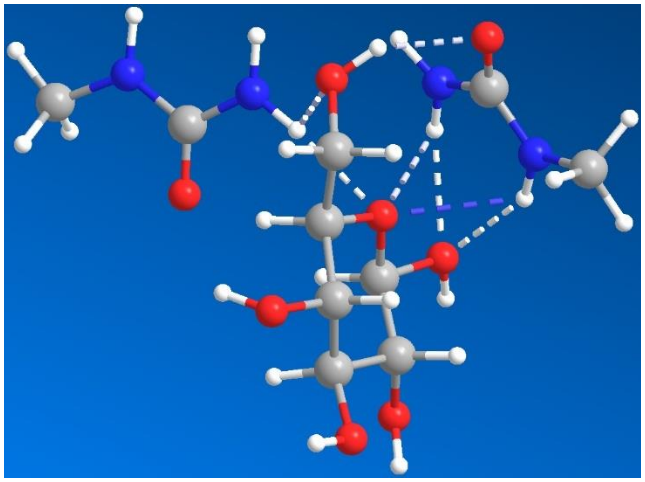

Figure S2-8. *HB analysis of DES 8, ChCl:Glc:U (1:1:1)*

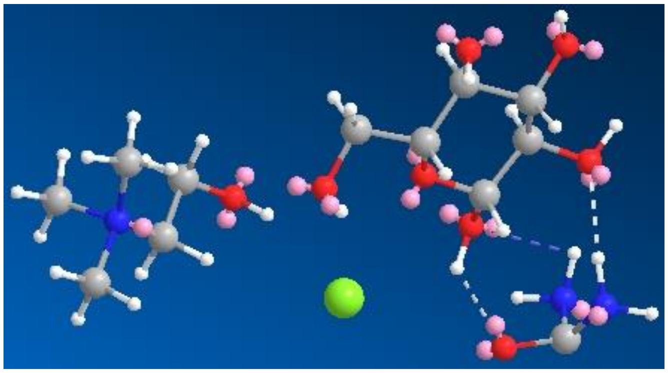

Figure S2-9. *HB analysis of DES 9, ChCl:Glc:U (1:1:2)*

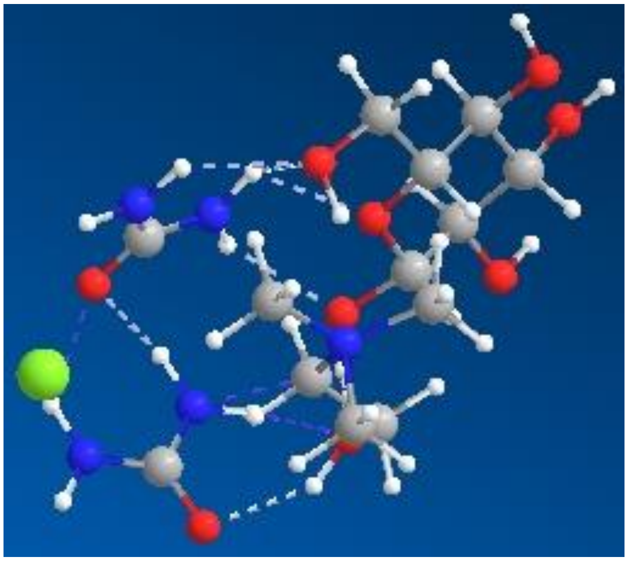

Figure S2-10. *HB analysis of DES 10, ChCl:MMH:U (1:0.5:2)*

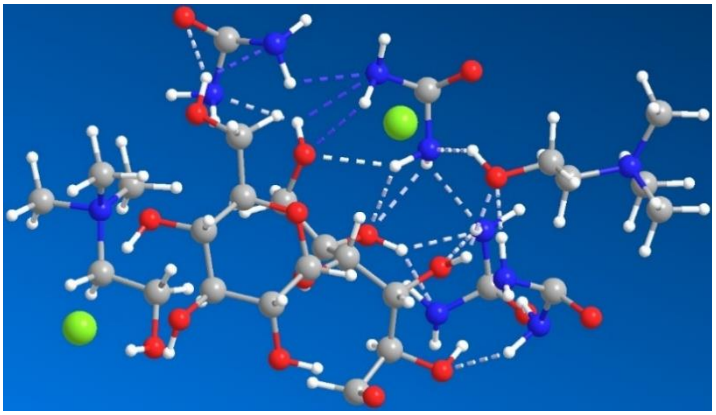

Figure S2-13. *HB analysis of DES 13, ChCl:Ara (1:1)*

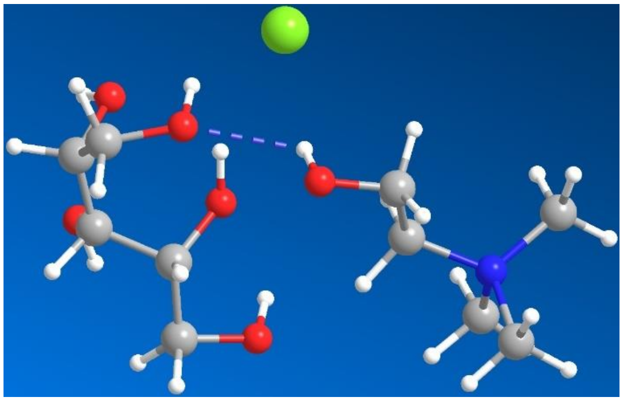

Figure S2-14. *HB analysis of DES 14, ChCl:Xyl (1:1)*

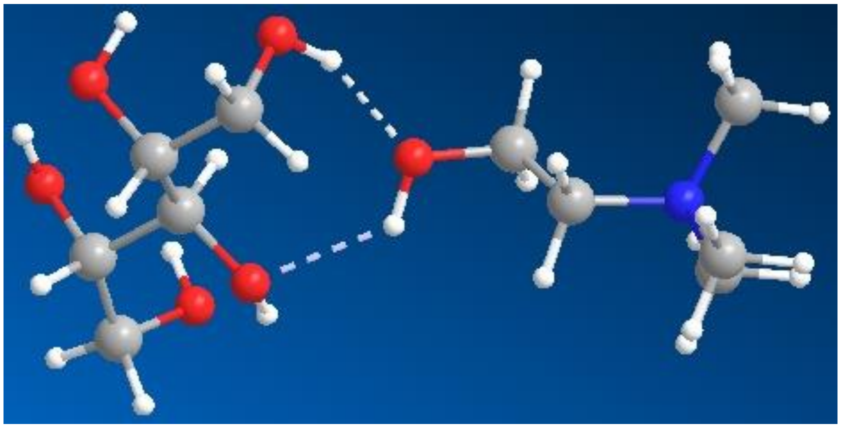

Figure S2-15. *HB analysis of DES 15, ChCl:Sorb (1:1)*

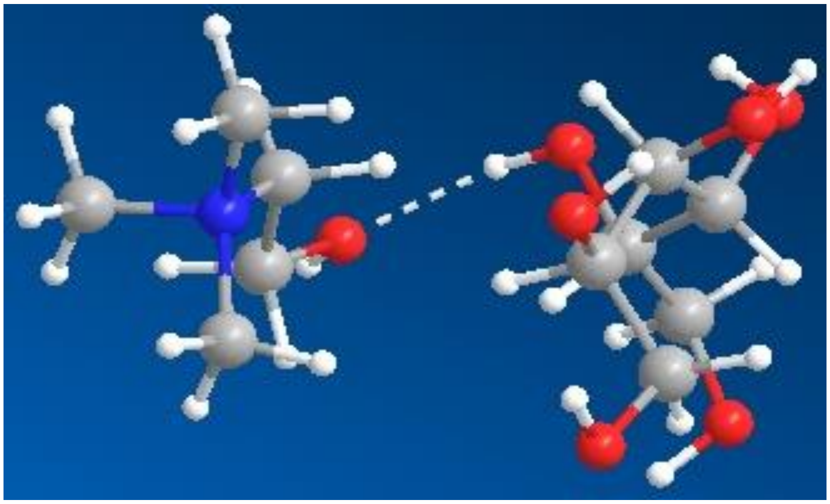

Figure S2-16. *HB analysis of DES 16, ChCl:Lact (2:1)*

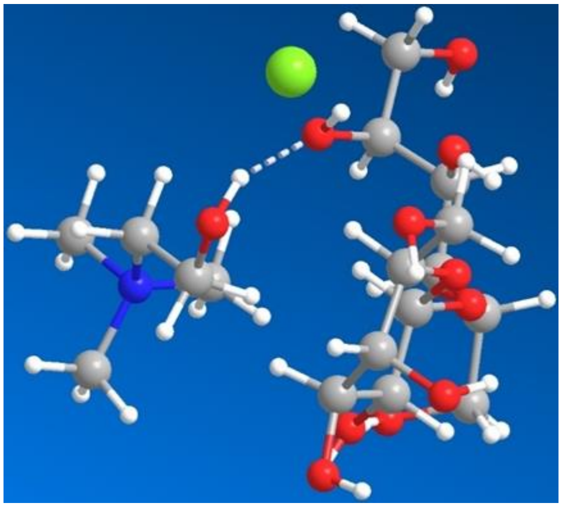

Table S1. Donors, acceptors and hydrogen bond length within the investigated R-NADES

DES	Donor	Acceptor	Bond length (Å)
DES 1	O (OH ChCl)	O (CH2OH glc)	2.390
	O (OH ChCl)	O (OH glc)	1.900
	O (OH glc)	O (OH ChCl)	2.321
DES 2	O (CH2-OH glc)	O (ChCl)	2.135
	O (H2O)	O (CH2OH glc)	2.330
	O (H2O)	O (CH2OH glc)	2.253
	O (H2O)	O (pyrane ring O)	1.963
DES 3	O (OH glc)	O (ChCl)	1.867
	O (OH ChCl_1)	O (H2O)	1.870
	O (OH ChCl_2)	O (OH glc)	1.928
DES 4	O (OH ChCl_1)	O (OH ChCl_2)	1.887
	O (OH ara)	O (OH ChCl_1)	1.922
DES 5	O (OH ChCl_1)	O (H2O 1)	1.999
	O (H2O 1)	O (OH maltose)	2.123
	O (OH maltose)	O (OH ChCl_2)	2.441
	O (OH maltose)	O (OH ChCl_3)	2.270
	O (OH ChCl_3)	O (H2O 3)	2.177
	O (H2O 4)	O (H2O 3)	1.881
	O (H2O 4)	O (H2O 2)	2.007
	O (H2O 3)	O (H2O 1)	2.049
	O (H2O 3)	O (H2O 2)	2.411
DES 6	N1 (NH2 urea_1)	O (OH glc)	2.237
	N1 (NH2 urea_1)	O (urea_2)	2.100
	N2 (NH2 urea_1)	O (urea_2)	2.111
	O (CH2OH glc)	O (urea_1)	2.018
	N (NH2 urea_2)	O (OH glc)	2.157
DES 7	N (NH2 urea_1)	O (pyrane ring O)	2.174
	N (NH2 urea_1)	O (CH2OH glc)	2.231
	N (NH2 urea_2)	O (pyrane ring O)	2.309
	N (NH2 urea_2)	O (OH glc)	2.190
	N (NH-Me urea_2)	O (pyrane ring O)	2.464
	N (NH-Me urea_2)	O (OH glc)	2.184
	O (CH2OH glc)	O (urea_2)	1.903
DES 8	N1 (NH2 urea)	O (OH glc)	2.078
	N2 (NH2 urea)	O (OH glc)	2.432
	O (OH glc)	O (urea)	2.002
DES 10	N (NH2 urea_1)	O (OH glc)	2.256
	N (NH2 urea_2)	O (pyrane ring O)	2.110
	O (OH glc)	O (ChCl)	1.925
	O (OH glc)	N (NH2 urea_2)	2.494
	N (NH2 urea_2)	O (ChCl)	2.211

	O (ChCl)	O (urea_2)	1.889
	N (NH2 urea_2)	O (urea_1)	2.047
	N (NH2 urea_1)	O (CH2OH glc)	2.196
	N (NH2 urea_1)	O (CH2OH glc)	2.177
DES 10	N (NH2(1) urea_1)	N (NH2 urea_2)	2.360
	N (NH2(2) urea_1)	O (CH2OH maltose)	2.147
	O (CH2OH maltose)	N (NH2(2) urea_1)	2.285
	O (CH2OH maltose)	O (urea_1)	2.120
	N (NH2(1) urea_2)	O (OH maltose)	2.081
	O (OH maltose)	N (NH2(1) urea_2)	2.373
	N (NH2(2) urea_2)	O (OH maltose)	2.193
	O (OH ChCl)	N (NH2(2) urea_2)	2.226
	N (NH2(1) urea_3)	O (OH cHcL_1)	2.121
	N (NH2(2) urea_3)	O (OH ChCl_1)	2.181
	N (NH2(1) urea_4)	O (OH maltose)	2.030
	O (OH maltose)	N1 (NH2 urea_4)	2.317
	N (NH2(2) urea_3)	N2 (NH2 urea_4)	2.240
	O (OH maltose)	N2 (NH2 urea_4)	2.308
	N (NH2(1) urea_3)	O (urea_4)	2.130
	N (NH2(1) urea_3)	O (OH maltose)	2.159
DES 13	O (OH ChCl)	O (OH arabitol)	2.284
DES 14	O (OH xyl)	O (ChCl)	1.890
	O (ChCl)	O (OH xyl)	2.173
DES 15	O (OH sorb)	O (ChCl)	1.903
DES 16	O (OH ChCl)	O (OH lactitol)	2.025

Figure S3. Thermograms, TG and DSC, of selected R-NADES.

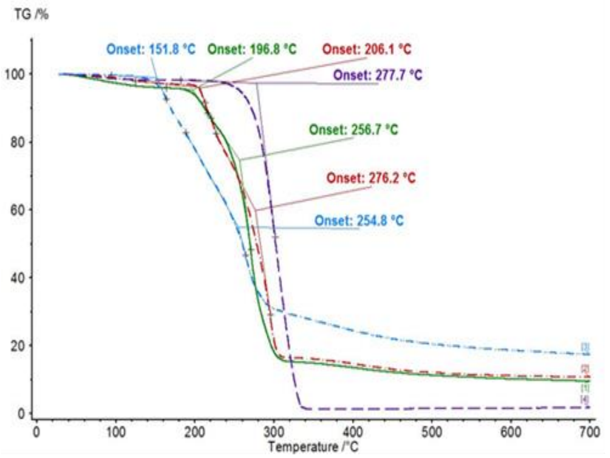

Fig. A1-A. Termograms for NADES: [1] ChCl:Glc:H_2O (2:1:1), [3] U:ChCl:Glc (2:1:1), [4] ChCl:sorbitol 1:1

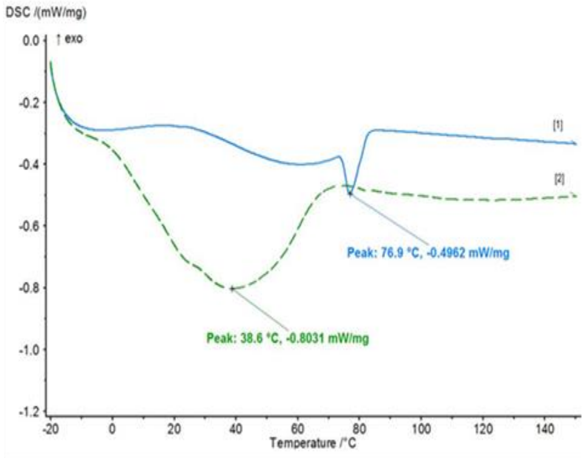

Fig. A1-B. DSC traces of NADES: [1] ChCl:Glc (2:1) 2:1 and [2] ChCl:Ara (1:1)

Figure S4. Thermogravimetric properties of R-NADES mixtures and their relationships

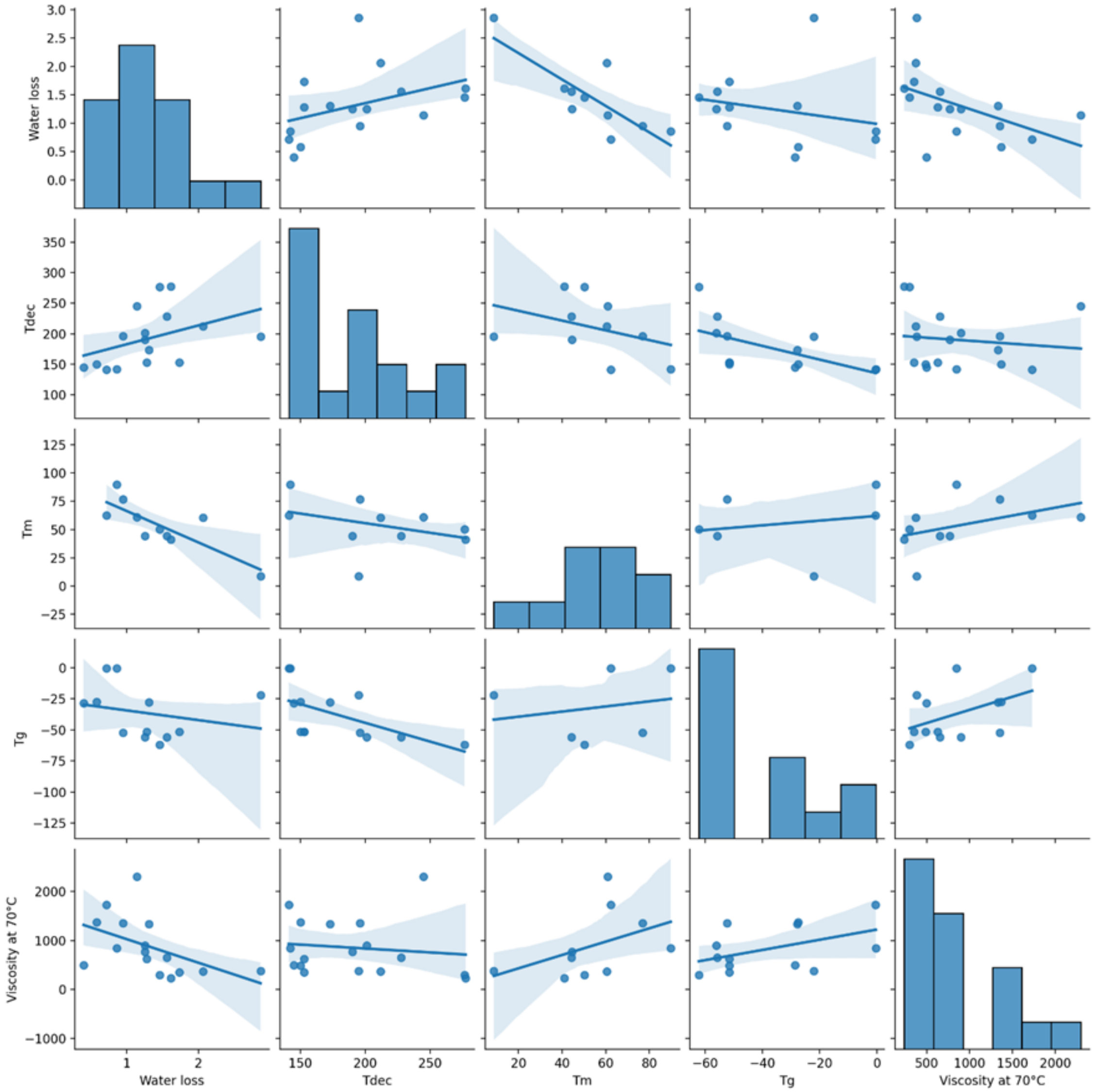

Figure S5: Graphic representation of the frontier molecular orbital HOMO and LUMO orbitals for R0NADES

Figure S5-1. *Distribution of HOMO orbitals (A) and LUMO orbitals (B) orbital for DES 1, ChCl:Glc (2:1)*

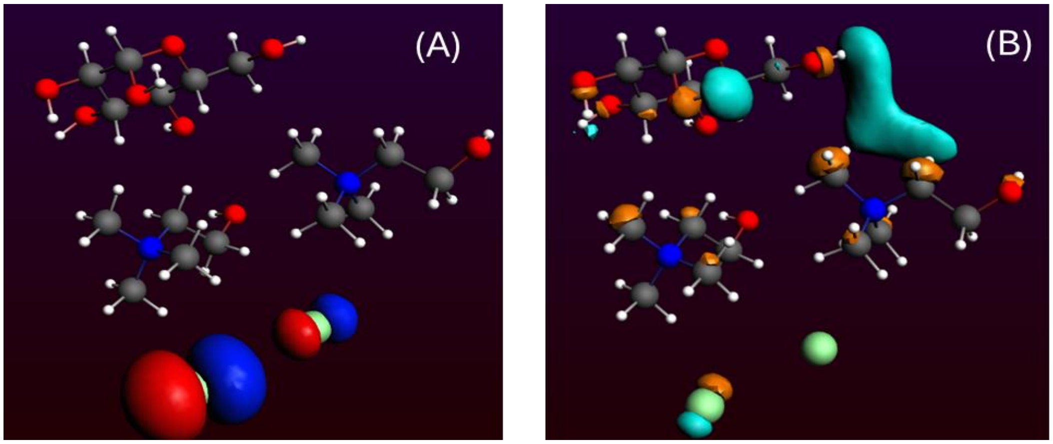

Figure S5-2. *Distribution of HOMO orbital (A) and LUMO orbital (B) for DES 2, ChCl:Glc:H_2O (1:1:1)*

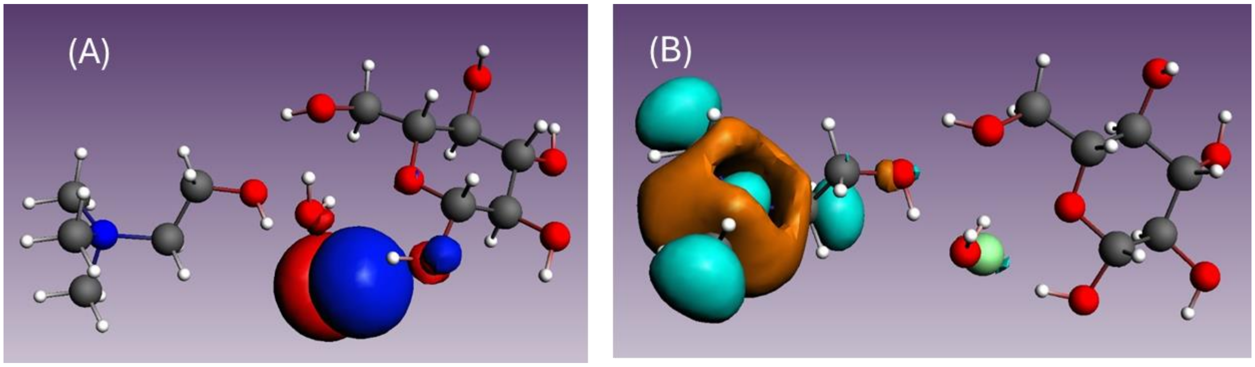

Figure S5-3. *HOMO orbital distribution for DES 3, ChCl:Glc:H_2O (2:1:1)*

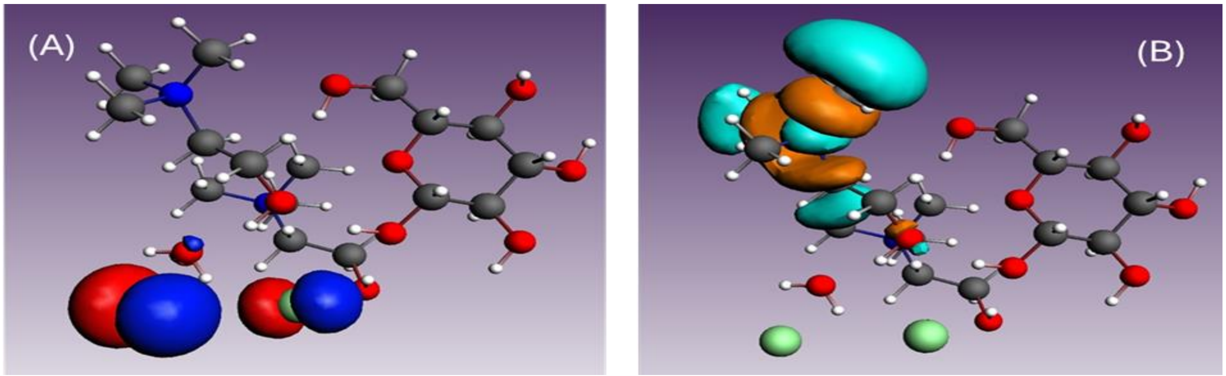

__Figure S5-4__. Distribution of HOMO orbital (A) and LUMO orbital (B) for DES 4, ChCl:Arabinose (2:1)

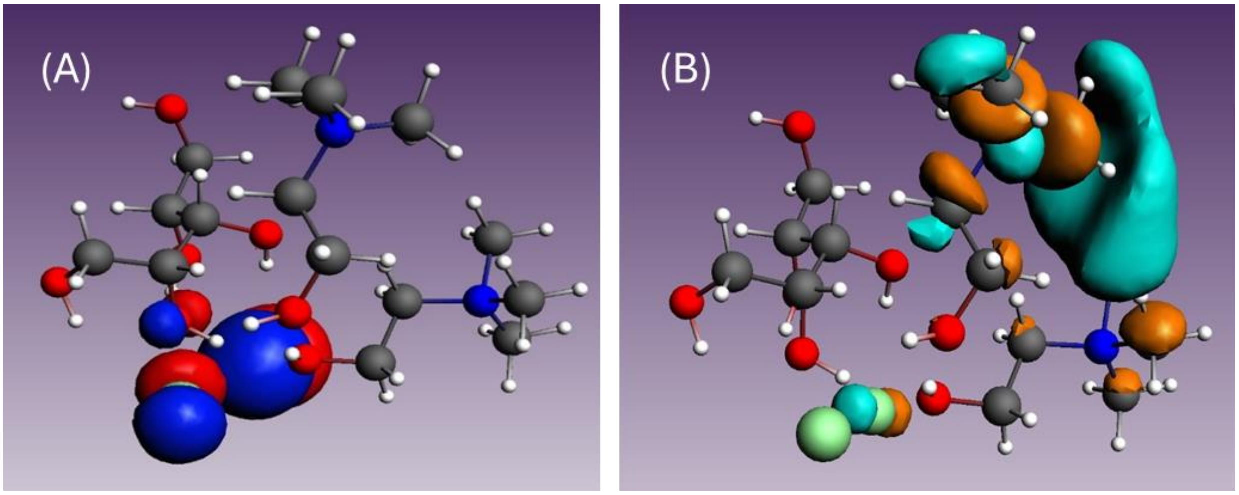

__Figure S5-5__. HOMO orbital distribution for DES 5, ChCl:MMH:H_2O (4:1:4)

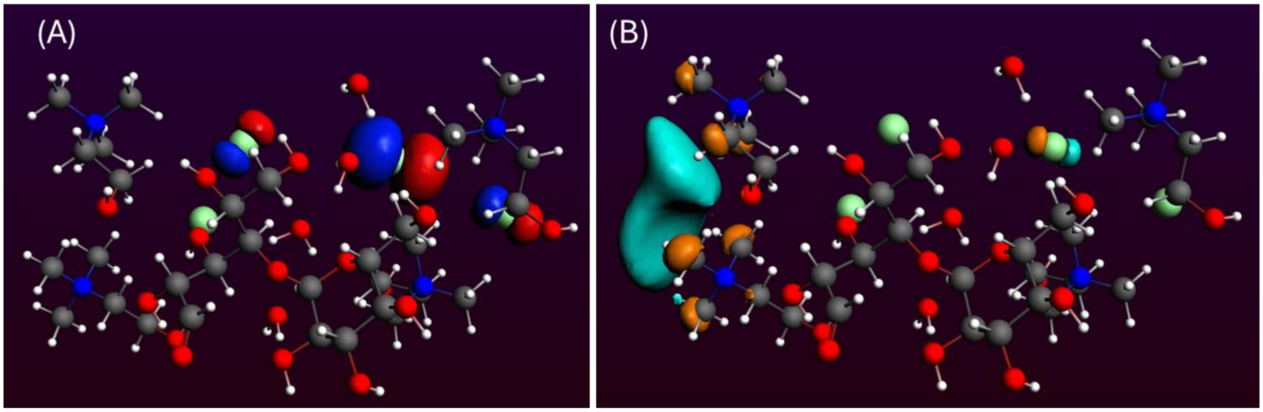

__Figure S5-6__. Distribution of (A) HOMO orbital and LUMO orbital (B) for DES 6, U:Glc (2:1)

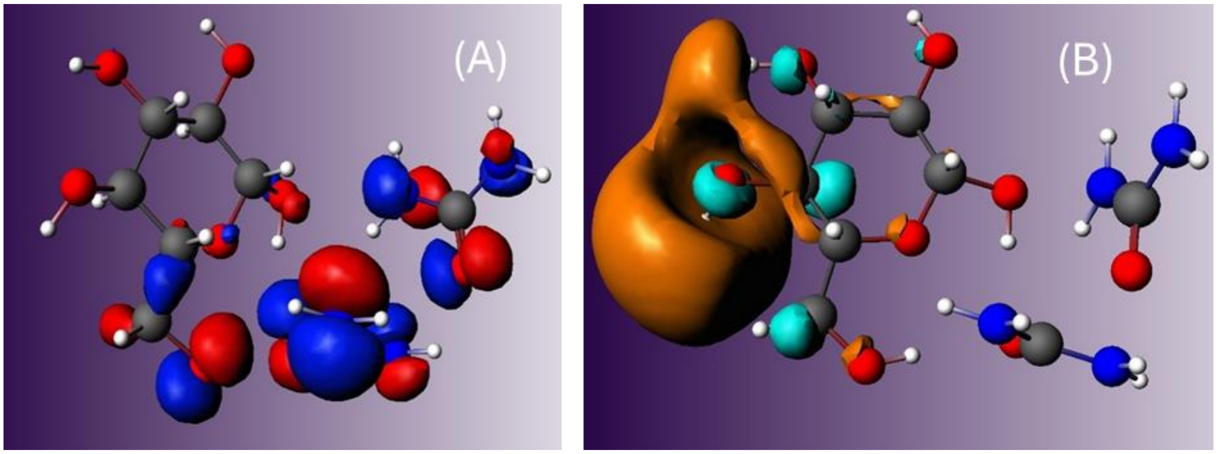

Figure S5-7. *Distribution of (A) HOMO orbital and LUMO orbital (B) for DES 7, MU:Glc (2:1)*

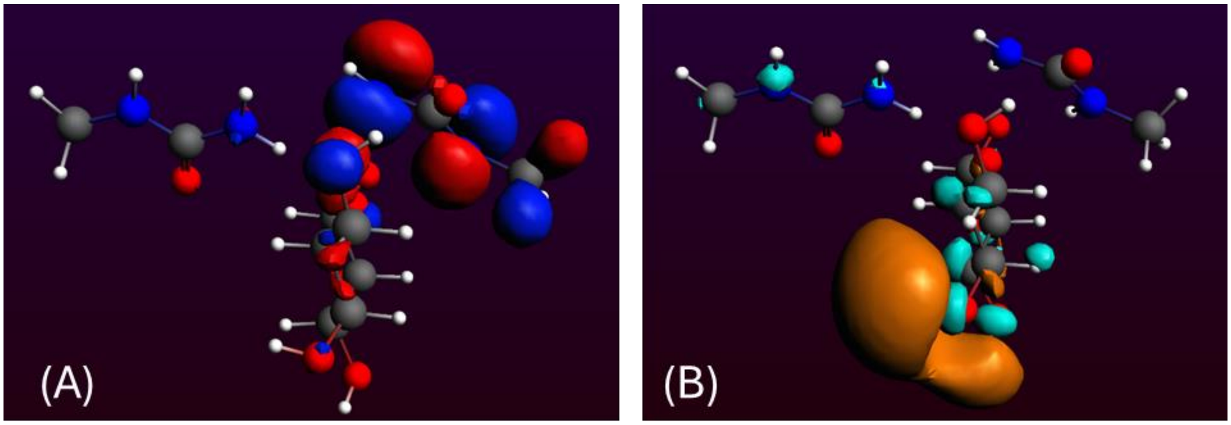

Figure S5-8. *Distribution of (A) HOMO orbital and LUMO orbital (B) for DES 8, ChCl:Glc:U (1:1:1)*

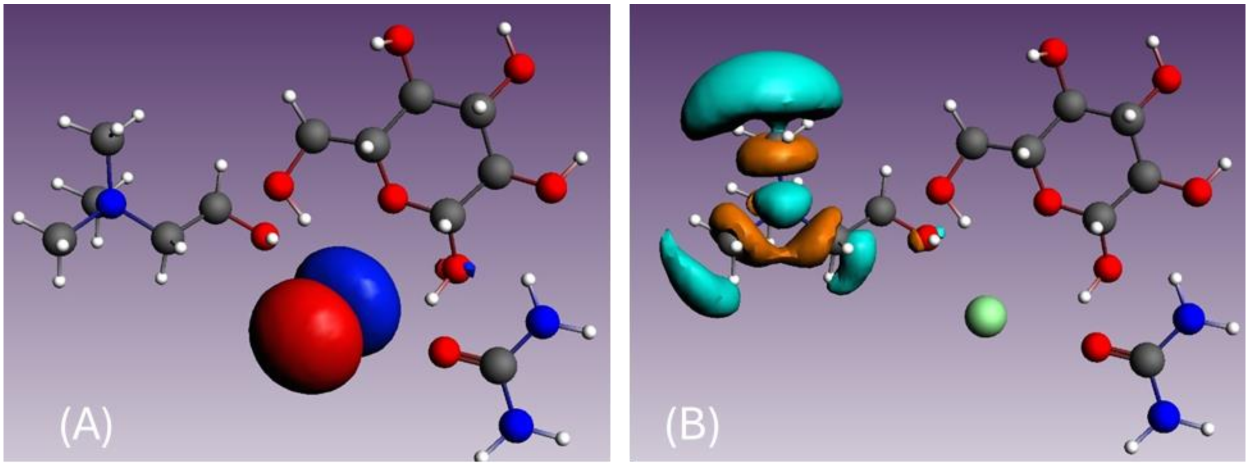

Figure S5-9. *Distribution of (A) HOMO orbital and LUMO orbital (B) for DES 9, ChCl:Glc:U (1:1:2)*

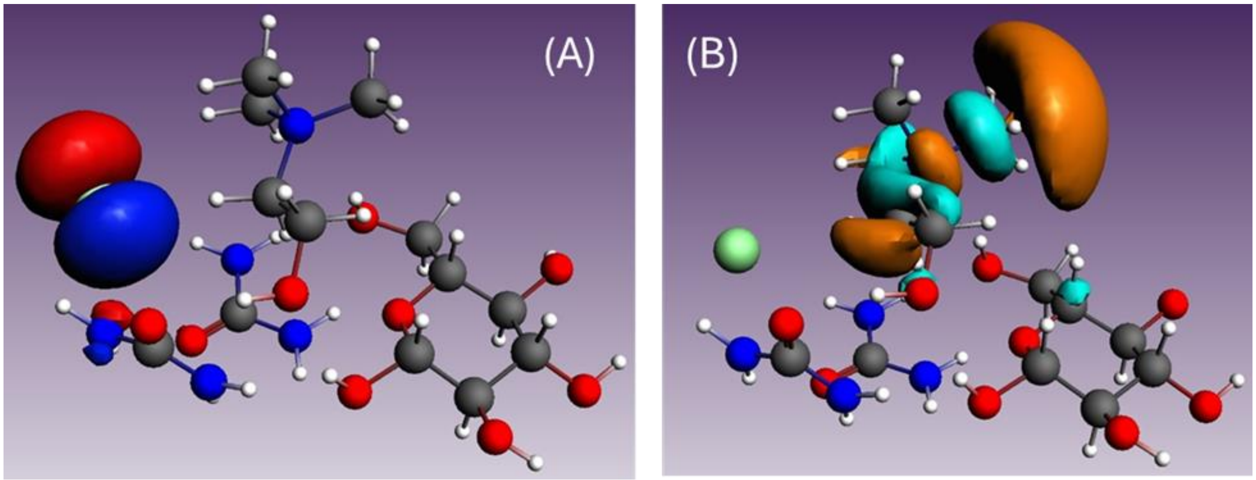

__Figure S5-10__. Distribution of (A) HOMO orbital and LUMO orbital (B), for DES 10 ChCl:MMH:U (1:0.5:2)

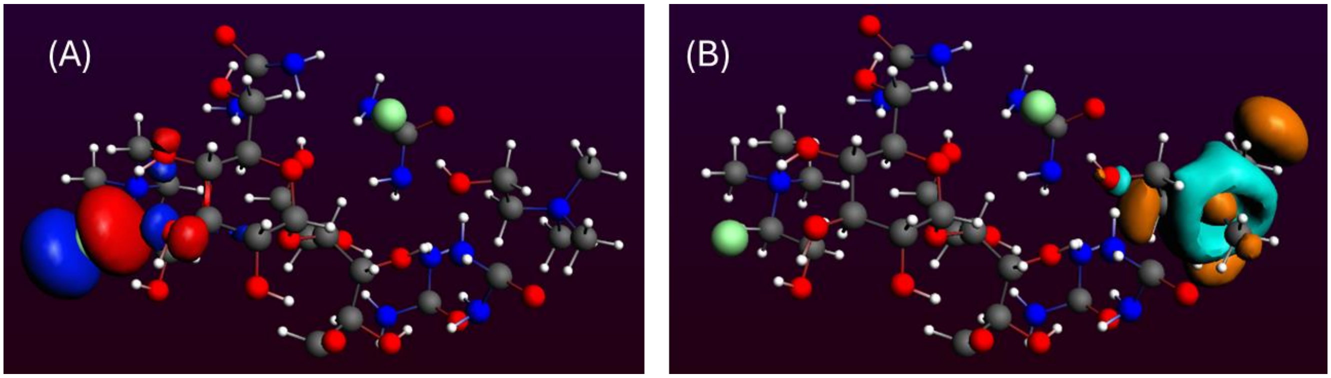

__Figure S5-13__. Distribution of (A) HOMO orbital and LUMO orbital (B) for DES 13, ChCl:Ara (1:1)

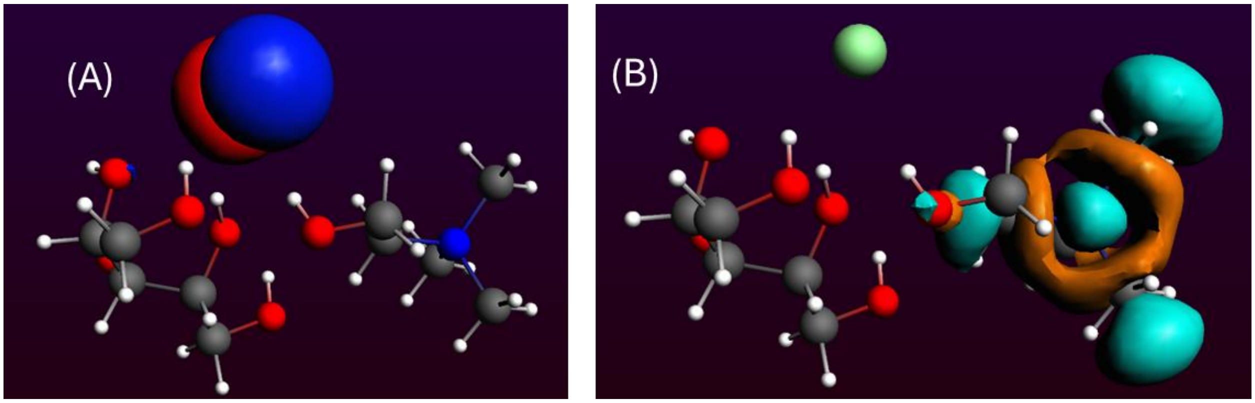

__Figure S5-14__. Distribution of (A) HOMO orbital and LUMO orbital (B) for DES 14, ChCl:Xyl (1:1)

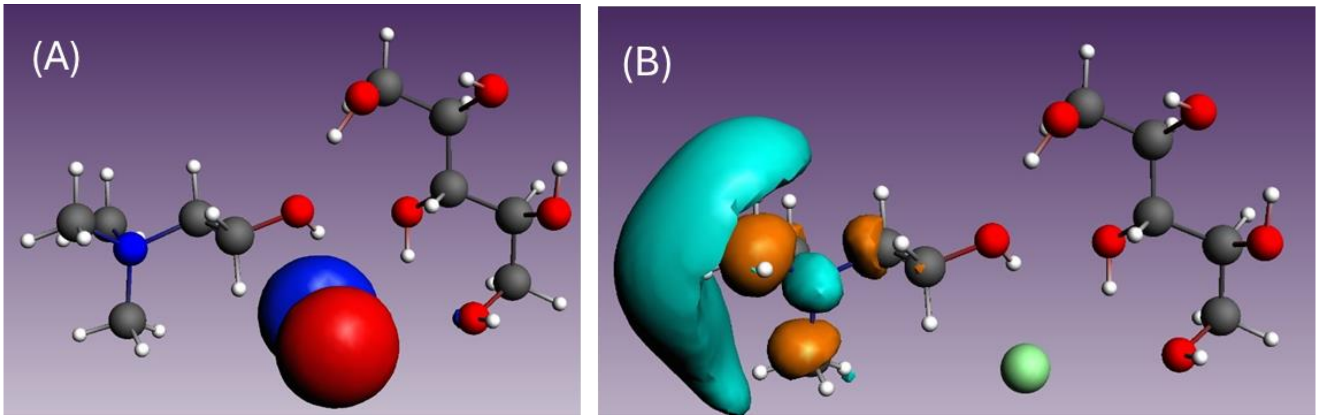

Figure 5-15. *Distribution of (A) HOMO orbital and LUMO orbital (B) for DES 15, ChCl:Sorb (1:1)*

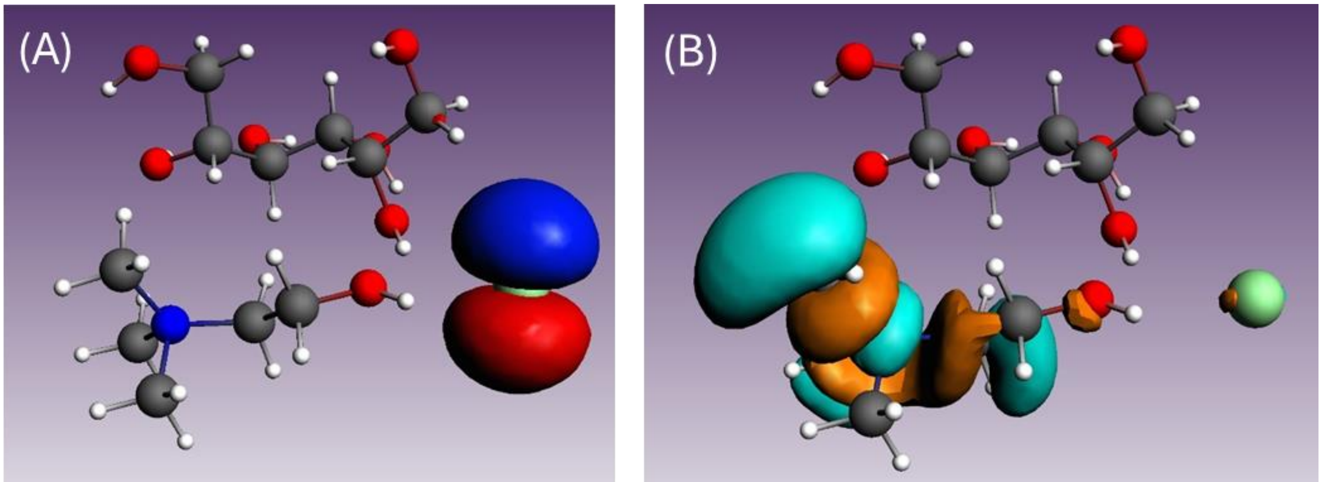

Figure S5-16. *Distribution of (A) HOMO orbital and LUMO orbital (B) for DES 16, ChCl:LMH (1:1)*

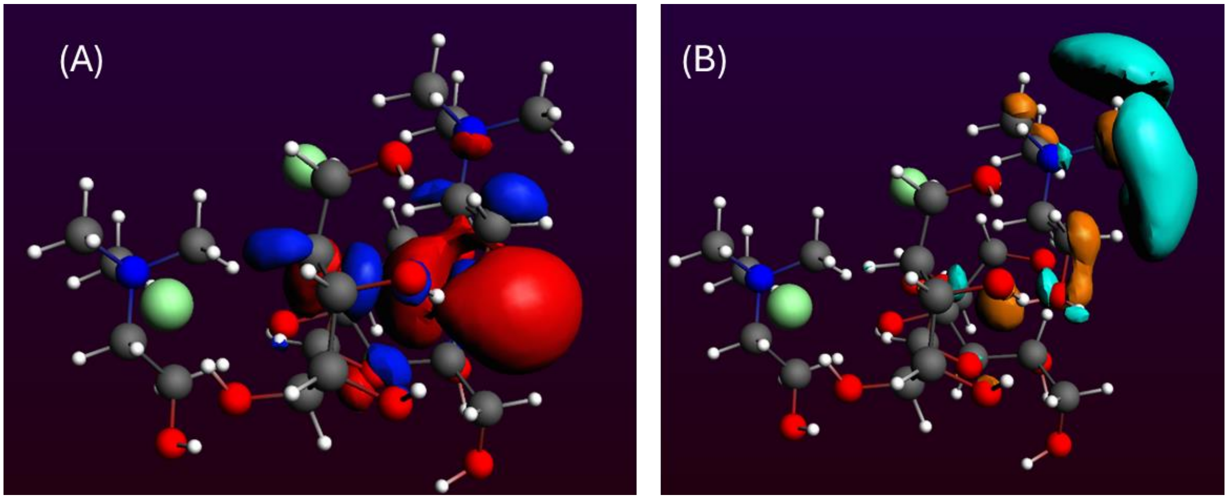

Table S2. Global reactivity descriptors of R-NADES.

R-NADES		Total energy (H)	μ (eV)	η (eV)	ω (eV)
Nr.	Composition				
1	ChCl:Glc (2:1)	-2.207	-2.207	0.037	65.822
2	ChCl:Glc:H_2O (1:1:1)	-2.675	-2.675	0.341	10.492
3	ChCl:Glc:H_2O (2:1:1)	-2.686	-2.686	0.208	17.343
4	ChCl:Arabose (2:1)	-2.585	-2.585	0.011	303.73
5	ChCl:MMH:H_2O (4:1:4)	-2.321	-2.321	0.004	673.68
6	U:Glc (2:1)	-3.346	-3.346	2.476	2.261
7	MU:Glc (2:1)	-3.010	-3.010	2.262	2.003
8	ChCl:Glc:U (1:1:1)	-2.417	-2.417	0.311	9.390
9	ChCl:Glc:U (1:1:2)	-2.658	-2.658	0.390	9.058
10	ChCl:MMH:U (1:0.5:2)	-2.952	-2.952	0.622	7.005
13	ChCl:D-Ara (1:1)	-2.876	-2.876	0.689	6.002
14	ChCl:Xyl (1:1)	-2.831	-2.831	0.846	4.737
15	ChCl:D-Sorb (1:1)	-2.796	-2.796	0.381	10.259
16	ChCl:LMH (2:1)	-2.645	-2.645	0.423	8.270
